# A P-type pentatricopeptide repeat-containing protein interacts with the Japanese soil-borne wheat mosaic virus movement protein and modulates susceptibility to infection

**DOI:** 10.1099/jgv.0.002260

**Published:** 2026-06-04

**Authors:** Claudia J. Strauch, Khalid Amari, Steffen Ostendorp, Ruth Veevers, Emmanuel Boutant, Nico Sprotte, Richard J. Morris, Julia Kehr, Annette Niehl

**Affiliations:** 1 Julius Kühn Institute (JKI) - Federal Research Centre for Cultivated Plants, Institute for Epidemiology and Pathogen Diagnostics, Messeweg 11-12, 38104 Brunswick, Germany; 2 Julius Kühn Institute (JKI) - Federal Research Centre for Cultivated Plants, Institute for Biosafety in Plant Biotechnology, Erwin-Baur-Str. 27, 06484 Quedlinburg, Germany; 3 Institute for Plant Science and Microbiology, Universität Hamburg, Ohnhorststr. 18, 22609 Hamburg, Germany; 4 Computational and Systems Biology, John Innes Centre, Norwich, UK; 5 Laboratory of Bioimaging and Pathologies, CNRS UMR 7021, Faculty of Pharmacy, University of Strasbourg, 74 Route du Rhin - CS 60024, F-67400, Illkirch, Strasbourg, France

**Keywords:** PPR protein, RNA immunoprecipitation, subcellular localization, target RNAs, viral movement protein, virus–host interaction

## Abstract

As obligate intracellular pathogens, plant viruses interact with host factors to achieve efficient replication and spread. Host factors also play important roles in virus detection and antiviral defence. Furoviruses cause substantial losses in cereals and efficient control measures are lacking. We therefore used Japanese soil-borne wheat mosaic furovirus movement protein (MP^JSBWMV^) in immunoprecipitation experiments to identify host interaction partners in the model host plant *Nicotiana benthamiana*. We identified a P-type pentatricopeptide repeat-containing protein (NbPPR) as the interaction partner of the MP^JSBWMV^. Fluorescent protein-tagged NbPPR localized to chloroplasts and to the cytosol and plasmodesmata (PD) depending on the location of the fluorescent protein tag, suggesting that the protein may be dually localized to these cellular compartments. MP^JSBWMV^:RFP and GFP:NbPPR colocalized and interacted at PD as shown by fluorescence resonance energy transfer fluorescence lifetime imaging and co-immunoprecipitation. To functionally characterize the MP^JSBWMV^-interacting NbPPR, we immunoprecipitated GFP:NbPPR-interacting RNAs and identified a motif overrepresented in the GFP:NbPPR-bound RNAs. The motif is consistent with the PPR target sequence predicted using PPR code. While most of the RNA targets encode subunits of the organellar NADH-dehydrogenase complex, we also identified RNAs encoding cell wall proteins. Downregulation of NbPPR, using virus-induced gene silencing and subsequent infection with fluorescent protein-tagged viruses, resulted in increased numbers of infection sites for JSBWMV in plants silenced for NbPPR compared to controls. We propose a model to explain how virus infection may affect NbPPR function and how NbPPR may regulate the infection cycle. As PPRs are increasingly explored for the modulation of gene expression, virus-targeting PPRs may be developed into tools for virus control in future.

## Introduction

The Japanese soil-borne wheat mosaic virus (JSBWMV, species *Furovirus japonicum*) belongs to the genus *Furovirus*, members of which can cause significant crop losses in susceptible plants [1]. JSBWMV is a positive-sense bipartite RNA virus with rigid rod-shaped particles [2]. The 7.2 kb long RNA1 encodes two versions of the RNA-dependent RNA polymerase involved in virus replication and the movement protein (MP), required for virus cell-to-cell spread [3,4]. The 3.5 kb long RNA2 encodes the capsid protein (CP) for encapsidation, a capsid protein read-through (CPRT) protein involved in virus transmission, a 25 K protein of unknown function and a cysteine-rich silencing suppressor [5,8]. Since its first discovery in Japan, it was found to infect barley as well as wheat [9]. Despite the name, JSBWMV isolates were also detected in barley in Germany and France [10,11]. JSBWMV is transmitted by the plasmodiophorid vector *Polymyxa graminis*, which forms resting spores containing infectious virus [12].

To better understand virus infection, knowledge of host factors regulating infection is crucial. Viral MPs are important multifunctional proteins involved in binding to viral genomes, shielding viral RNA from recognition by the host, interfering with defence responses, modulating transmission, transporting the viral genome within and between cells and interacting with plasmodesmata (PD), the intercellular communication channels between plant cells [13,16]. Thus, studying host interaction partners of viral MPs may illuminate mechanisms involved in these pathways. The MP of JSBWMV belongs to the 30K MP family [4,17]. MP^JSBWMV^ localizes to PD and to plasma membrane (PM) microdomains and can reestablish the movement of a movement-deficient tobacco mosaic virus (TMV) [18]. Furthermore, it was shown that MP^JSBWMV^ can bind plant and viral RNA.

With respect to host interaction partners, the MP of the JSBWMV-related furovirus Chinese wheat mosaic virus (CWMV, species *Furovirus chinense*) interacted with a pectin methylesterase in the cell wall of *Nicotiana benthamiana* leaves [19]. CWMV replicase interacted with the *N. benthamiana* heat shock protein 70 [20], a protein often identified as an interaction partner of viral proteins [21,22]. CP^CWMV^ interacted with cytosolic glyceraldehyde‐3-phosphate dehydrogenase, a negative regulator of autophagy, and the autophagy‐related protein 3, a key factor in autophagy [23]. Serine/threonine protein kinase osmotic stress/ABA-activated protein kinase 7 was shown to interact and phosphorylate the CWMV cysteine-rich protein (CRP^CWMV^) and promote CWMV infection [24]. Additionally, RNA-binding UBP1-associated protein 2C (UBA2C) was found to interact with CRP^CWMV^. UBA2C was shown to regulate cell death and H_2_O_2_ production, and silencing of UBA2C significantly enhanced susceptibility to the virus in wheat [24]. A role for chloroplasts in defence against CWMV was supported by a proteomics study. This study found 390 differentially abundant proteins upon CWMV infection in *N. benthamiana*, of which 218 proteins were distributed to the chloroplasts [25]. Notably, 35.62% of the upregulated and 66.94% of the downregulated proteins were localized in the chloroplast [25].

Pentatricopeptide repeat-containing proteins (PPRs) are a family of RNA-binding proteins present mainly in eukaryotes, but the family is greatly expanded in land plants with over 500 members in *Nicotiana attenuata* [26]. PPR proteins are defined through a P-motif with a length of over 35 aa. The PPR family is further divided into two subclasses, the P-class and the PLS-class according to the presence of L (long variant of P-motif) and S (short variant of P-motif) domains [27]. PPR proteins are involved in post-transcriptional processing mainly in organelles, i.e. in mitochondria and/or chloroplasts, and participate in RNA C- to-U editing, RNA stabilization, RNA cleavage and splicing and the regulation of RNA translation [26,28,30]. Some PPR proteins were shown to have functions outside organelles. Because PPR proteins are central to organelle function, their loss impacts plant growth and development as well as responses to stress. In particular, P-class PPR proteins are involved in processing and stabilization of 5′ and 3′ termini of target RNAs in organelles by acting as a barrier for exoribonucleases through binding specific RNA sequences [28]. To regulate translation, PPRs may bind to 5′UTRs and activate translation by unmasking the ribosome binding site or inhibit translation by binding to the ribosome binding site [30]. In mitochondria, PPRs can also be components of ribosomes and act as homing factors to find mitochondrial RNAs and initiate translation [31].

To identify host proteins important for furovirus infection, we conducted immunoprecipitation (IP) of overexpressed MP^JSBWMV^:GFP in the JSBWMV host plant *N. benthamiana* and identified a PPR protein as an MP interaction partner. To understand the role of the NbPPR (for *N. benthamiana* PPR) during virus infection, we investigated its subcellular localization and RNA-binding properties and identified a possible target RNA motif for this NbPPR protein. Moreover, using virus-induced gene silencing (VIGS), we analyzed the function of NbPPR during infection and spread of JSBWMV and the unrelated turnip mosaic potyvirus (TuMV).

## Methods

### Plant material

*N. benthamiana* plants were grown in the greenhouse at 20–24 °C with 16 h light and 8 h dark cycles and at least 200 W m^−^² light intensity. Plants were maintained under these conditions for agroinoculation or infection with tobacco rattle virus (TRV, species *Tobravirus tabaci*) VIGS vectors and TuMV-GFP. Plants infected with JSBWMV-CPRT:RFP were kept at 17 °C with 16 h light and 8 h dark cycles and at least 200 W m^−^² light intensity.

### Viruses

The GenBank accession numbers for JSBWMV strain JT are NC_038850.1 (RNA1) and NC_038851.1 (RNA2) [32]. pYL192 (TRV1) and pYL156 (TRV RNA2) were a gift from Savithramma Dinesh-Kumar (Addgene plasmid # 148968; http://n2t.net/addgene:148968; RRID: Addgene_148968), and PYL192 and pYL156 are based on the TRV Ppk20 strain with the GenBank accession numbers AF406990 and Z36974. The entire pTRV2 sequence is deposited in GenBank with the accession number AF406991 [33]. TuMV:GFP derived from strain UK1 [34]; GenBank accession number EF028235.1 was used.

### Fluorescent protein-tagged fusion proteins

MP^JSBWMV^:GFP and MP^JSBWMV^:RFP, GFP, pGWB455 (free RFP, cytoplasmic marker) and P19 were published before [18,35, 36]. NbPPR:GFP, NbPPR:RFP and GFP:NbPPR binary vectors were generated by GATEWAY cloning (Invitrogen, Thermo Fisher Scientific, Waltham, USA) according to the manufacturer’s instructions [37]. RNA from *N. benthamiana* young leaves was extracted using the NucleoSpin RNA Plant and Fungi Kit (Macherey-Nagel, Düren, Germany), and cDNA synthesis was carried out with the ProtoScript® II Reverse Transcriptase Kit [New England Biolabs (NEB), Ipswich, USA], according to the manufacturer’s instructions. The ORF of *NbPPR* (Niben101Scf06603g01015.1) was PCR-amplified from *N. benthamiana* cDNA and recombined in pDONR™/Zeo (Invitrogen). For the construction of NbPPR:GFP and NbPPR:RFP, the destination vectors pB7FWG2 or pB7RWG2 were used, respectively. For the construction of GFP:NbPPR, the destination vector pB7WGF2 was used [37]. Sequences of entry and expression vectors were verified via DNA sequencing. All primers are depicted in Table S1 (available in the online Supplementary Material).

### Transient protein expression and microscopy

For transient expression in *N. benthamiana*, GFP and P19 were transformed into agrobacterium strain C58C1, while all other constructs were in agrobacterium strain GV3101. Agrobacterium transformation of *N. benthamiana* was performed as described [18]. For staining with aniline blue, a solution containing the dye (67 mM sodium phosphate, 0.5–1% aniline blue) was vacuum infiltrated into leaf discs. After 10 min in a dark place, the samples were analysed by confocal microscopy. Microscopy at a Leica DM6 microscope platform (Leica Microsystems, Wetzlar, Germany) was performed as described [18], with the following detection ranges: aniline blue (420–480 nm), GFP (495–555 nm), RFP (590–630 nm) and chloroplast (660–720 nm). All constructs were observed in different leaf samples under the different co-expression conditions.

### Identification of MP^JSBWMV^-interacting partners by MS

MP^JSBWMV^:GFP or GFP was expressed in leaves of *N. benthamiana* for 3 days via agroinoculation. Leaves were then used for co-IP using GFP-trap magnetic agarose beads. MS was performed as described in [38]. In short, the immunoprecipitated proteins were separated by SDS-PAGE and stained with colloidal Coomassie [39]. Bands differing between control and MP^JSBWMV^:GFP-containing samples were excised, and in-gel digestion was performed as described with minor modifications [40]. Excised bands were transferred to siliconized 0.5 ml tubes (Eppendorf, Hamburg, Germany) and subsequently destained for 1 h in 50% (v/v) acetonitrile and 50% (v/v) ammonium bicarbonate (50 mM, NH_4_HCO_3_). The gel pieces were then dehydrated with 100% acetonitrile for 10 min at room temperature. The acetonitrile was removed prior to trypsin digestion. Trypsin digestion was performed by adding 0.01 µg µl^−1^ modified porcine trypsin (Promega, Madison, USA) in ammonium bicarbonate (50 mM) to the gel pieces and overnight incubation at 37 °C. The supernatant was applied directly to MALDI-MS. Using AnchorChip™ Targets (Bruker Daltonics GmbH, Bremen, Germany), the sample and matrix preparation were accomplished according to the manufacturer’s instructions. The acquisition of spectra was done on a Bruker Ultraflex III MALDI-TOF/TOF mass spectrometer (Bruker Daltonics GmbH) operated in positive reflectron mode. For analysing the positively charged ions, the m/z range of 600–4000 Da was used. Spectra analysis was done using mMass [41]. For protein identification, the MASCOT data search engine (http://www.matrixscience.com [42]) was used with the following search settings: 0.3 Da peptide tolerance, one permitted miscleavage and methionine oxidation as a variable modification. Searches were carried out against the non-redundant database of the NCBI [43]. The search was limited to Viridiplantae. If protein coverage of >25% and a significant MASCOT score were achieved, proteins were considered identified. The UniProtKB protein database [44] was used to blast protein hits with unknown function to find homologous proteins with known function. MS/MS ion search on selected peptides was performed if more than one protein was identified in one spot by MALDI-MS to validate additional proteins. Single peptides were set as parent ions. The fragmentation was then either done by post-source decay or by additionally enabling collision-induced decay utilizing nitrogen as collision gas. The analysis of the fragments was performed with mMass using MS/MS ion search. The settings of the precursor peptide tolerance and MS/MS tolerance were adjusted to 0.3. Further settings were identical to the peptide mass fingerprint analyses. If at least three peptides could be assigned, a protein was considered identified.

### Comparison of PPR sequences from *Nicotiana* species

Pentatricopeptide repeat-containing protein At1g62910-like (XP_009608994.1) *Nicotiana tomentosiformis* was identified in MS. Protein sequence was used to identify the homologue gene in *N. benthamiana* using blast in the solgenomics *N. benthamiana* draft genome (https://solgenomics.net, last retrieval date 15 September 2022 [45,48]). Protein sequence of NbPPR (Niben101Scf06603g01015.1) was used to search the UniProt database (https://www.uniprot.org [49]) to identify close homologues in *Nicotiana tabacum* (XM_016652926.1) and *Nicotiana sylvestris* (A0A1U7VCB8) representing the pentatricopeptide repeat-containing protein At1g63150-like. Sequence alignment was performed with CLC main workbench version 23.0.1.

### Co-IP

GFP or RFP-tagged proteins were expressed in *N. benthamiana* leaves for 3 days. Leaves were used in IP with GFP-Trap® magnetic agarose beads (Chromotek, Proteintech, Chicago, USA). The IPs were performed according to the manufacturer’s protocol with minor modifications. Lysis was achieved by grinding the leaf material with liquid N_2_ and subsequent addition of 1 vol (v/v) lysis buffer [50 mM TRIS-HCl (pH 7.5); 150 mM NaCl; 2.5 mM MgCl_2_; 0.5% Nonidet® P40 (Substitute) BioChemica (AppliChem GmbH, Darmstadt, Hessen); 1× Protease Inhibitor cocktail (cOmplete Tablets Mini, EDTA-free; Roche, Basel, Switzerland)]. After 30-min incubation on ice, the lysate was cleared with filtration through one layer of Miracloth. The cleared lysate was added to the GFP-Trap® magnetic agarose beads (equilibrated according to the manufacturer’s instructions) and incubated for 1 h at 4 °C end-over-end. Washing was performed by magnetic separation of the beads and resuspending three times in 500 µl wash buffer [50 mM TRIS-HCl (pH 7.5); 150 mM NaCl; 2.5 mM MgCl_2_; 0.5% Nonidet® P40 (Substitute) BioChemica; 1× Protease Inhibitor cocktail (cOmplete Tablets Mini, EDTA-free)]. The proteins were eluted by adding 2× SDS sample buffer and boiling for 10 min at 94 °C. To confirm the interaction identified by MS, IPs with co-expressed proteins were conducted.

### Protein detection in Western blots

SDS sample buffer [120 mM TRIS-HCl (pH 6.8), 20% glycerol, 4% SDS, 0.04% bromophenol blue and 10% *β*-mercaptoethanol] was added to plant samples or precipitated proteins from IP and heated for 10 min at 95 °C prior to loading of the samples onto an SDS gel. SDS-PAGE was performed with a 12% separating gel and a 6% stacking gel followed by electroblotting onto an ImmobilonP PVDF membrane (0.45 µm pore size, Millipore, Merck, Sigma-Aldrich, St. Louis, USA). To detect GFP, anti-GFP polyclonal antibody (PA1-980A, Thermo Fisher Scientific) was used as the primary antibody. Goat anti-Rabbit IgG (H+L) conjugated to horseradish peroxidase was used as a secondary antibody (Thermo Fisher Scientific). To detect RFP, anti-RFP monoclonal antibody (RF5R, Thermo Fisher Scientific) and the F(ab′)2-Goat anti-Mouse IgG (H+L) secondary antibody conjugated to horseradish peroxidase (Thermo Fisher Scientific) were used. To determine protein sizes the PageRuler™ Plus Prestained Protein Ladder, 10 bis 250 kDa (Thermo Fisher Scientific) was used.

### RNA immunoprecipitation and RNA sequencing

RNA immunoprecipitation (RIP) was performed as published [18]. IP and subsequent RNA-seq was conducted in two repetitions per condition with pooled samples consisting of leaves from independent plants. GFP:NbPPR or GFP was expressed for 3 days in the leaves of *N. benthamiana*. During sampling, a formaldehyde fixation was applied, in which 1% formaldehyde solution was infiltrated into leaves. This was followed by a washing step with infiltration of glycine solution (125 nM). After rinsing the leaves with ice-cold dH_2_O, the leaves were frozen in liquid nitrogen. Five hundred milligrams of ground leaf material was used as starting material for RIP with GFP-Trap® magnetic agarose beads (Chromotek). IP was conducted according to the manufacturer’s protocol with minor changes. The lysis buffer contained 50 mM TRIS-HCl (pH 7.5); 150 mM NaCl, 2.5 mM MgCl_2_ and 0.5% Nonidet® P40 (Substitute) BioChemica (AppliChem GmbH); 1× Protease Inhibitor cocktail (cOmplete Tablets Mini, EDTA-free; Roche); 50 U ml^−1^ RNase inhibitor (murine, NEB); and 0.5 mM DTT. To the ground leaf material, 1 vol (v/v) lysis buffer was added and the mixture was incubated for 30 min on ice. Followed by filtration through one layer of Miracloth, the cleared lysate was added to 30 µl GFP-Trap® magnetic agarose beads (equilibrated according to the manufacturer’s instructions). After 1-h end-to-end incubation at 4 °C, the beads were washed by magnetic separation and resuspension in lysis buffer three times. For RNA elution from the beads, TRI Reagent® (Sigma-Aldrich) was added to the beads. RNA extraction with TRI Reagent® was performed according to the manufacturer’s instructions. Total RNA-seq was performed on an Illumina NovaSeq platform (Genewiz, Azenta, Chelmsford, USA) with a depth of 10 million read pairs per sample in a 2×150 bp configuration. The GFP samples served as controls in [18]. Bioinformatic analysis was performed for two replicates of GFP:NbPPR and GFP RIP, respectively. Bioinformatic analysis was performed using the Galaxy platform [50,51]. Adapters were removed and trimming (reads shorter than 20 nt) was conducted with TrimGalore. The *N. benthamiana* reference dataset (https://solgenomics.net/) was used to map the reads. Default settings were used allowing three mismatches during alignment using the ‘hierarchical indexing for spliced alignment of transcripts’ (HISAT) programme [52]. Reads were counted with Samtools idxstats [53]. To identify common and specific transcripts among the different treatments, a Venn diagram comparison [54] was used. The log2 fold change between GFP:NbPPR replicates and GFP replicates was calculated using counts per million for each replicate. Enriched RNAs with a fold change over 2 and at least 100 reads in the GFP:NbPPR RIP were used for further interpretation (Table 1).

**Table 1.** List of RNAs bound to GFP:NbPPR RNAs which were at least twofold enriched in the GFP:NbPPR RIP compared to the GFP RIP with at least 100 reads in the two GFP:NbPPR RIP replicates are depicted. Several *N. benthamiana* scaffolds are annotated with the same annotation. The used *N. benthamiana* draft genome also contained chloroplastic and mitochondrial sequences. The reads and normalized reads representing the sequencing depth for each of the RNAs per replicate and condition analyzed are depicted in Table S6.

Annotation	Annotation solgenomics	Annotation *N. benthamiana* draft genome (scaffold)	Fold change	No. of reads	Description
Mitochondrial protein, putative (*Medicago truncatula*)	ref|XP_003588355.1|	Niben101Scf04451g00027.1	2.07	1973	Unknown function
NADH-quinone oxidoreductase subunit H	sp|Q5FGW5|NUOH_EHRRG	Niben101Scf05689g02012.1	2.62	2480	Protein is part of the NADH:ubiquinone oxidoreductase complex which plays part in electron shuttling [87,88]
Conserved hypothetical protein (*Ricinus communis*) gb|EEF25773.1| conserved hypothetical protein (*Ricinus communis*)	ref|XP_002536610.1|	Niben101Scf15885g00035.1	2.59	2667	Unknown function
		Niben101Scf02309g03017.1	2.31	1350	
		Niben101Scf09696g01037.1	2.21	241	
Protein IQ-DOMAIN 14 (IQD 14)	sp|Q8LPG9|IQD14_ARATH	Niben101Scf01681g02009.1	2.22	986	IQD 14 localizes to the microtubule in *A. thaliana* and *N. benthamiana* and plays a role in plant organ development [89,90]
NADH-quinone oxidoreductase subunit H 1	sp|Q3J3F1|NUOH1_RHOS4	Niben101Scf08162g00010.1	2.81	2044	Protein is part of the NADH:ubiquinone oxidoreductase complex which plays part in electron shuttling [87,88]
Conserved hypothetical protein (*Ricinus communis*) gb|EEF45986.1| conserved hypothetical protein (*Ricinus communis*)	ref|XP_002516369.1|	Niben101Scf04432g06003.1	2.04	1426	Unknown function
		Niben101Scf05540g00010.1	2.18	1005	
NADH-quinone oxidoreductase subunit H	sp|Q3YS40|NUOH_EHRCJ	Niben101Scf11347g00005.1	2.31	1311	Protein is part of the NADH:ubiquinone oxidoreductase complex which plays part in electron shuttling with roles in respiratory chain complexes [87,88]
orf109 (mitochondrion) (*Panax ginseng*) gb|AHJ81034.1| orf109 (mitochondrion) (*Panax ginseng*)	ref|YP_009091709.1|	Niben101Scf04048g00015.1	2.58	1598	Unknown function
		Niben101Scf00367g07004.1	2.93	877	
		Niben101Scf06928g00013.1	3.13	687	
		Niben101Scf14482g00003.1	3.06	137	
Unknown protein		Niben101Scf00854g06020.1	2.17	730	Unknown function
Hero resistance protein 3 homologue (*Solanum lycopersicum*)	emb|CAD29727.1|	Niben101Scf01184g09019.1	2.01	476	Hero resistance protein 3 homologue was described in the context of nematode resistance in potato [91]
NADH dehydrogenase subunit 2 (mitochondrion) (*Isoetes engelmannii*)	gb|ADT62143.1|	Niben101Scf02074g06077.1	2.12	296	Core subunit of the mitochondrial membrane respiratory chain NADH dehydrogenase (Complex I) and functions in the transfer of electrons [92]
Ribosomal protein S10 (*Medicago truncatula*)	ref|XP_003588337.1|	Niben101Scf02074g01039.1	2.29	326	Ribosomal protein S10 belongs to the small ribosomal subunit, ribosomes catalyse mRNA-directed protein synthesis [93,94]
NADH-quinone oxidoreductase subunit D	sp|Q135×7|NUOD_RHOPS	Niben101Scf03515g00078.1	2.25	309	NADH-quinone oxidoreductase is a large and complex redox proton pump with roles in respiratory chain complexes [92]
Unknown protein		Niben101Scf00916g01010.1	2.04	188	Unknown function
Unknown protein		Niben101Scf00482g08002.1	2.36	496	Unknown function
DNA polymerase (mitochondrion) (*Silene vulgaris*)	gb|AFI44305.1|	Niben101Scf14427g00008.1	2.08	272	Belongs to the DNA-directed DNA polymerase family B in mitochondria [95]
Unknown protein		Niben101Scf05250g00010.1	2.18	207	Unknown function
BnaUnng03880D, partial (*Brassica napus*)	emb|CDY70488.1|	Niben101Scf03515g00058.1	2.35	308	Unknown function
Chitinase 8	sp|Q7XCK6|CHI8_ORYSJ	Niben101Scf02041g00002.1	2.23	204	Chitinases are enzymes that catalyse the hydrolysis of the beta-1,4-*N*-acetyl-d-glucosamine linkages in chitin polymers [96]
NADH-ubiquinone oxidoreductase chain 4	sp|Q9ZZ45|NU4M_SQUAC	Niben101Scf00849g01019.1	2.08	228	Core subunit of the mitochondrial membrane respiratory chain NADH dehydrogenase (Complex I) and functions in the transfer of electrons [92]
NADH-quinone oxidoreductase subunit H	sp|B3CUK2|NUOH_ORITI	Niben101Scf03431g00004.1	2.02	215	Protein is part of the NADH:ubiquinone oxidoreductase complex which plays part in electron shuttling [87,88]
NADH-quinone oxidoreductase subunit D	sp|Q11JK3|NUOD_CHESB	Niben101Scf18129g00003.1	2.38	183	NADH-quinone oxidoreductase is a large and complex redox proton pump with roles in respiratory chain complexes [92]
NADH-ubiquinone oxidoreductase chain, putative (*Ricinus communis*) gb|EEF27701.1| NADH-ubiquinone oxidoreductase chain, putative (*Ricinus communis*)	ref|XP_002534687.1|	Niben101Scf10881g00025.1	2.20	248	Core subunit of the mitochondrial membrane respiratory chain NADH dehydrogenase (Complex I) and functions in the transfer of electrons [92]
BnaCnng48510D (*Brassica napu*s)	emb|CDY65732.1|	Niben101Scf14482g00006.1	2.12	119	Unknown function
		Niben101Scf18129g00016.1	3.11	255	
Cytochrome c biogenesis FN (mitochondrion) (*Hevea brasiliensis*)	dbj|BAO50886.1|	Niben101Scf08015g02002.1	2.10	157	Cytochrome c biogenesis FN is involved in the biogenesis of c-type cytochromes. Cytochromes C are electron-transfer proteins [97]
orf116d (mitochondrion) (Batis maritima) gb|AIC83424.1| orf116d (mitochondrion) (*Batis maritima*)	ref|YP_009045821.1|	Niben101Scf05439g02038.1	2.41	125	Unknown function
NADH dehydrogenase subunit 5 (mitochondrion) (*Silene vulgaris*)	gb|AFI44276.1|	Niben101Scf04114g01046.1	2.07	141	Core subunit of the mitochondrial membrane respiratory chain NADH dehydrogenase (Complex I) and functions in the transfer of electrons [92]
4-Coumarate--CoA ligase-like 7 (*Morus notabilis*) gb|EXC31783.1| 4-coumarate--CoA ligase-like 7 (*Morus notabilis*)	ref|XP_010111818.1|	Niben101Scf03431g00012.1	2.12	194	Proteins have coenzyme A-ligase activity and take part in phenylpropanoid biosynthetic pathways [98]
orf34 gene product (mitochondrion) (*Daucus carota* subsp. *sativus*) gb|AEY81172.1| orf34 (mitochondrion) (*Daucus carota* subsp. *sativus*)	ref|YP_006291803.1|	Niben101Scf00853g03001.1	2.10	152	Unknown function
Germin-like protein subfamily 3 member 3	sp|P94072|GL33_ARATH	Niben101Scf02749g04008.1	2.32	178	Germin-like proteins are assumed to participate in the production of H_2_O_2_ [99]
Cytochrome C assembly protein LENGTH=256	AT2G07771.2	Niben101Scf01374g02004.1	2.76	214	Protein involved in the aggregation, arrangement and bonding together of a cytochrome complex; a cytochrome complex takes part in catalysis of redox reactions [100,101]
		Niben101Scf01745g07016.1	2.86	230	
		Niben101Scf02497g07001.1	2.53	157	
		Niben101Scf00485g00013.1	2.67	190	
		Niben101Scf02002g01024.1	3.03	224	
Cysteine-rich venom protein	sp|A6MFK9|CRVP_DEMVE	Niben101Scf00107g03008.1	2.90	153	Cysteine-rich secretory protein has toxin activity which interferes with the function of ion channels [102]
NADH-quinone oxidoreductase subunit D	sp|Q0APY7|NUOD_MARMM	Niben101Scf15885g00031.1	2.41	109	NADH-quinone oxidoreductase is a large and complex redox proton pump with roles in respiratory chain complexes [92]
30S ribosomal protein S3	sp|Q1GPA5|RS3_SPHAL	Niben101Scf00914g03002.1	2.44	152	Ribosomal protein S10 belongs to the small ribosomal subunit, ribosomes catalyse mRNA-directed protein synthesis [93,94]

### Prediction of RNA-binding motif

The RNA-seq data were used to predict a possible RNA-binding motif in NbPPR. A total of 168 RNAs with a fold change over 2 and at least 10 reads were used; additionally, 4 RNAs which were abundant in the GFP:NbPPR RIP (more than 20 reads) but not in the GFP control RIP were used for the prediction (Table S2). The XSTREME tool [55] within the MEME Suite [56] was used to identify the enriched motif. The 171 RNA sequences bound to GFP:NbPPR were used as the primary sequences, while all 2433 RNA sequences found in the RNA-seq data were used as the background sequences. The motif was identified using the *de novo* motif discovery algorithm STREME [55] which is executed within XSTREME. Search for the RNA-binding motif in viral sequences was performed with CLC main workbench version 23.0.1 with the motif search tool.

In a complementary approach, PPR code (https://biolib.com/YaoYinYing/pprcode/, last visited 25 August 2025 [57]) was used to predict the RNA target sequence of NbPPR (Niben101Scf06603g01015.1) using the ‘PPR finder’ setting (Table S3 summarizes the identified P-motif positions).

### Fluorescence resonance energy transfer–fluorescence lifetime imaging microscopy

Fluorescence resonance energy transfer (FRET)–fluorescence lifetime imaging microscopy (FLIM) was performed as described by [58,59] with little modifications [18]. To summarize, fluorescent proteins were expressed in *N. benthamiana* epidermal leaf cells for 2 to 5 days. Time-correlated single-photon counting FLIM measurements were accomplished with a home-built two-photon system. FRET-efficiency values above 5% were considered positive, indicating that protein-protein interaction occurred between donor and acceptor. A total of 90 and 218 measurements were taken per condition. Measurements were derived from 9 and 18 microscopy images, respectively, of leaf samples expressing GFP:NbPPR and GFP:NbPPR +MP^JSBWMV^:RFP. Significant differences were calculated according to Student’s t-test.

### Virus-induced gene silencing

VIGS was performed with TRV as a vector [33]. TRV RNA2 was modified to insert a 299 nt long part of the coding sequence of *NbPPR*. The *NbPPR* sequence was PCR-amplified from *N. benthamiana* cDNA, and restriction sites for Kpn1 and Xba1 were added. cDNA was synthesized with the ProtoScript® II Reverse Transcriptase Kit (NEB) with RNA from young *N. benthamiana* leaves that was extracted using the NucleoSpin RNA Plant and Fungi Kit (Macherey-Nagel), according to the manufacturer’s instructions. PCR fragment and TRV-RNA2 vector were digested with Kpn1 (NEB) and Xba1 (NEB), followed by purification with the NucleoSpin Gel and PCR Clean-up kit (Macherey-Nagel), according to the manufacturer’s instructions. Via a ligation, the *NbPPR* fragment was incorporated into the TRV-RNA2 vector and multiplied in *Escherichia coli* MC1061 cells. DNA sequence was verified with Sanger sequencing. TRV-RNA1 (unmodified), TRV-RNA2 : 00 (unmodified) and TRV-RNA2:NbPPR were transformed into agrobacteria GV3101. Agrobacteria with TRV-RNA1 and TRV-RNA2 : 00 or TRV-RNA2:NbPPR (respectively) were mixed equally and infiltrated into leaves of 2-week-old *N. benthamiana* with an OD of 0.5 each. Systemically infected leaves were then used to propagate the virus constructs. For virus inoculation (TRV:00, TRV:NbPPR), systemically infected *N. benthamiana* leaves from plants infected for at least 1 week were used to prepare a crude leaf extract containing celite and potassium-phosphate buffer (0.1 M K_2_HPO_4_ and KH_2_PO_4_ mix to pH 7). The crude leaf extract was rub-inoculated onto leaves of 2- to 4-week-old *N. benthamiana* plants. The leaves were rinsed with water after 2 min. To determine downregulation of *NbPPR* by TRV-VIGS over time, *NbPPR* transcript levels and TRV RNA levels were analysed in RNA obtained from systemically infected leaves at 10, 15 and 20 days post-infection (dpi) in three (15 dpi and 20 dpi) or four (10 dpi) independent experiments with three (15 dpi and 20 dpi) or four to eight (10 dpi) biological replicates by quantitative real-time PCR (see below). Statistical differences between the sample groups were tested with two-way ANOVA with a Bonferroni multiple comparison test.

At 10 dpi with TRV-VIGS constructs, TuMV-GFP or JSBWMV-CPRT:RFP, respectively, were rub-inoculated onto systemic, TRV:NbPPR or TRV:00 infected leaves. TuMV-GFP was inoculated as crude extract from infected leaves and infection with JSBWMV-CPRT:RFP was performed with infectious RNA [60]. Three (JSBWMV-CPRT:RFP) or four (TuMV-GFP) independent replicates each containing at least five (JSBWMV-CPRT:RFP) or at least two (TuMV-GFP) different leaves per treatment were analysed at six (JSBWMV-CPRT:RFP) or three (TuMV-GFP) and four (TuMV-GFP) dpi (Table S4). TuMV-GFP and JSBWMV-CPRT:RFP infection sites, which formed on the leaves, were observed with a Nikon SMZ25 fluorescence stereomicroscope (Nikon, Minato, Japan) with a bandpass filter for GFP and RFP. Area of the infection sites was determined with ImageJ 1.53c (http://imagej.nih.gov/ij, Java 1.8.0_172 (64-bit), Wayne Rasband, National Institutes of Health, USA). Two-way ANOVA with Bonferroni multiple comparison test was applied to investigate statistical significance. Significant differences between infection site numbers were investigated from the numbers of fluorescent JSBWMV-CPRT:RFP and TuMV-GFP infection sites formed on NbPPR silenced and control leaves. Welch’s test was used to compare the mean infection site numbers obtained upon fluorescent virus infection in NbPPR silenced compared to control conditions, and the Mann–Whitney U test was used to compare infection site numbers formed per leaf for each fluorescent virus in NbPPR silenced compared to control conditions. Statistical analyses and graph design were performed with BioRender Graph.

### Quantitative real-time reverse transcription polymerase chain reaction (RT-PCR)

To analyze *NbPPR* and TRV RNA levels, total RNA was extracted with TRI Reagent® (Sigma-Aldrich), followed by DNase I digestion with the TURBO DNA-free™ Kit (Invitrogen). cDNA synthesis was performed using the ProtoScript® II Reverse Transcriptase Kit (NEB) with random primers. All steps were conducted according to the manufacturer’s instructions. Quantitative real-time PCR was performed with the SYBR-green-based LUNA® universal qPCR Master Mix Kit (NEB) on a qTower 2.2 (Analytic Jena, Jena, Germany) and the analysis was done with the software qPCRsoft3.1 (RealTime PCR Application). Relative expression values depict 2^-ΔCT^ values. To obtain ΔCT values, CT was normalized to the CT values obtained for the housekeeping genes EF1-alpha and GAPDH. Primers are shown in Table S1 and published in [61].

## Results

### MP^JSBWMV^ interacts with an NbPPR protein

**Fig. 1.**
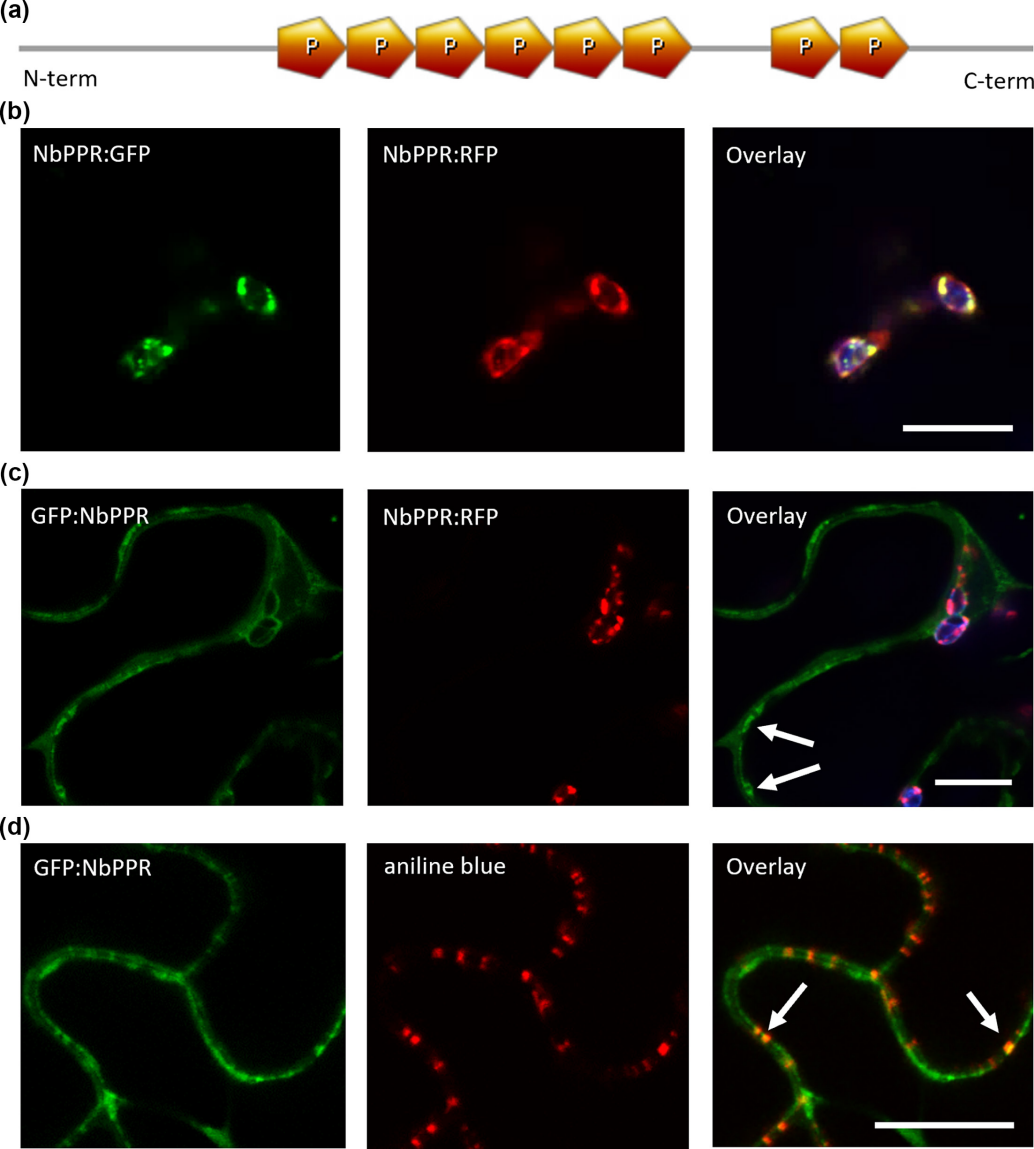
NbPPR is a P-class PPR-protein localized to chloroplasts or the cytoplasm/PD, depending on the localization of the fluorescent protein tag. (**a**) Schematic drawing of the NbPPR domain structure. Eight P-motifs were found in the NbPPR sequence using the ppr.plantenergy-website. The P-motifs are located in two blocks in the NbPPR-sequence; detailed positions of the P-motifs can be found in Table S3. Graphical position of P-motifs was drawn with the prosite.expasydrawing tool. (**b–d**) *N. benthamiana* epidermal cells co-expressing fusion proteins, single section images were taken two to five days post-infiltration of agrobacteria. Scale bars are 10 µm. (**b**) NbPPR:GFP (green, left panel) and NbPPR:RFP (red, central panel), localize to the chloroplast as an overlay with chloroplast autofluorescence (blue, right panel) shows. (**c**) GFP:NbPPR (green, left panel) localizes to the cytoplasm and PD and does not co-localize with NbPPR:RFP (red, central panel) as the overlay shows (right panel) (**d**) GFP:NbPPR (green, left panel) in aniline blue-stained cells (red, central panel) localizes partially at PD as seen in the overlay (right panel).

To find interaction partners of MP^JSBWMV^, we conducted co-IP experiments with MP^JSBWMV^:GFP, which was previously shown to be functional to support virus cell-to-cell movement [18]. In these experiments, the pentatricopeptide repeat proteinAt1g62910-like, accession number XP_009608994.1, was identified as a possible interaction partner (Fig. S1A, B). Using the blastp function on the solgenomics website and the *N. benthamiana* draft genome, we found that the closest orthologue was an undescribed sequence (Niben101Scf06603g01015.1) with 89.0% identity, here further referred to as NbPPR. Using the UniProt website, we identified close orthologues also in *N. tabacum* and *N. sylvestris* with 94.2% identity to the sequence of NbPPR (Fig. S1C). Using PPR.plantenergy (https://ppr.plantenergy.uwa.edu.au/, last visited 25 August 2025 [62,64]), eight P-motifs were found in the NbPPR sequence, identifying the NbPPR as a P-subclass PPR protein (Fig. 1a). Using TargetP2.0 (https://services.healthtech.dtu.dk/services/TargetP-2.0/, last visited 25 August 2025 [65]), the NbPPR sequence was predicted to contain a chloroplast transfer peptide in the N-terminal 60 amino acids with 55.25% probability (Fig. S1D, E).

Fluorescence microscopy experiments with leaves transiently co-expressing NbPPR fused C-terminally to GFP and RFP confirmed chloroplastic localization as indicated by co-localization with chloroplast autofluorescence (Fig. 1b). NbPPR:GFP and NbPPR:RFP formed granular structures inside chloroplasts, which perfectly colocalized. However, when NbPPR was fused N-terminally to GFP, a different localization pattern was observed. GFP:NbPPR localized to the cytoplasm and punctate structures at the cell periphery (Fig. 1c) which were identified as PD by co-localization with aniline blue-stained callose at PD (Fig. 1d). To validate that both proteins were expressed as fusion proteins, we conducted western blots with antibodies against the fluorescent protein tag. The observed protein sizes matched the predicted size for the NbPPR fusion proteins (Fig. S1F).

**Fig. 2.**
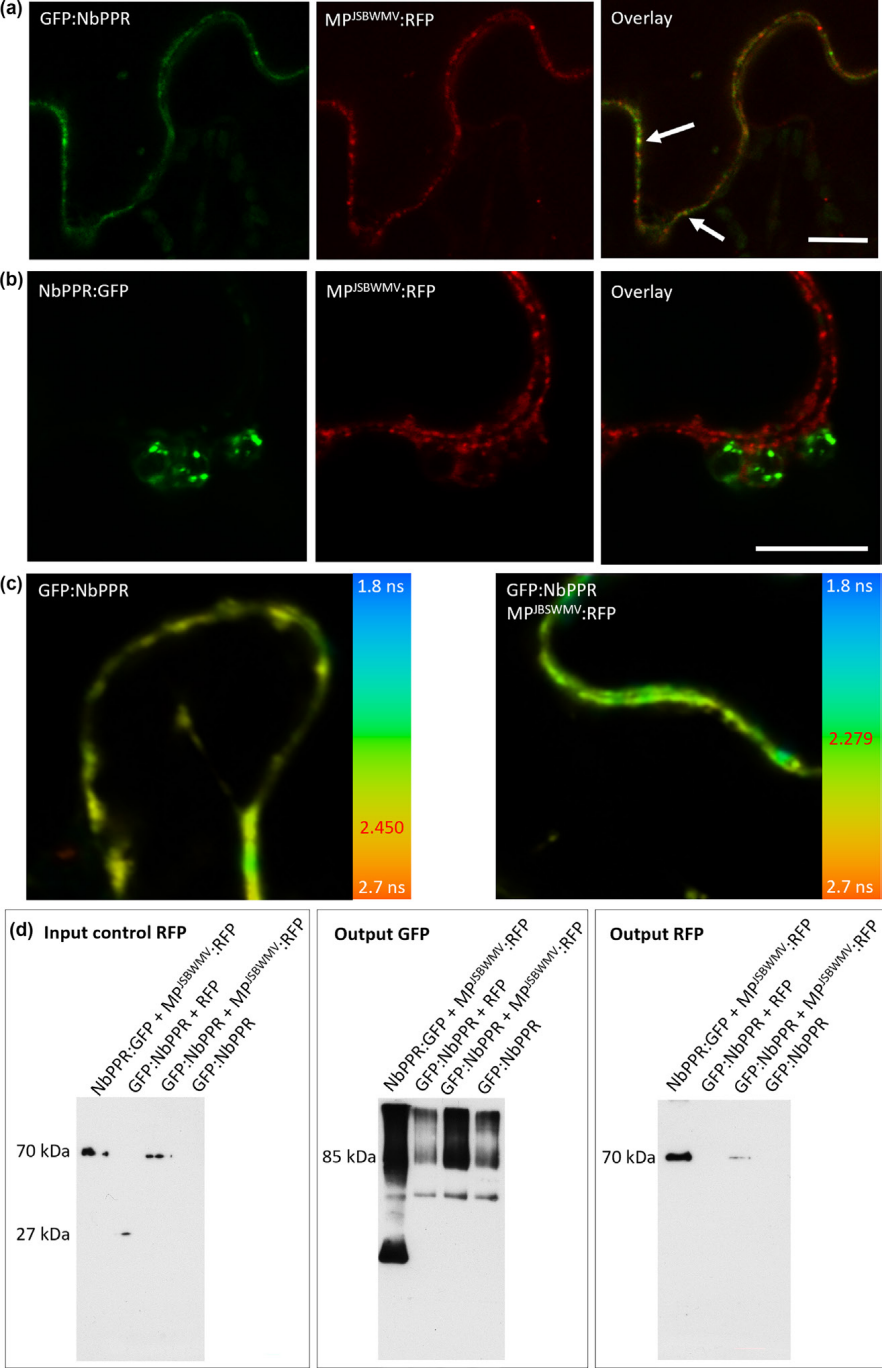
Characterization of NbPPR localization relative to MP^JSBWMV^ and confirmation of interaction between NbPPR and MP^JSBWMV^. (**a**) GFP:NbPPR (green, left panel) and MP^JSBWMV^:RFP (red, central panel) co-localize at PD in epidermal cells of *N. benthamiana* as seen in the overlay (right panel). Scale bar is 10 µm. (**b**) NbPPR:GFP (green, left panel) localizes to chloroplasts, while MP^JSBWMV^:RFP (red, central panel) localizes to PD and PM microdomains. Overlay (right panel) shows no overlapping fluorescence. (**c**) Fluorescence lifetime of GFP:NbPPR expressed in *N. benthamiana* epidermal cells was measured by FRET-FLIM. The images show fluorescence lifetime in a pseudo-colour scheme, ranging from 1.8 ns (blue) to 2.7 ns (orange) as shown in the colour-coded bar at the right hand side. The fluorescence lifetime of GFP:NbPPR expressed alone was 2.45 ns (left panel) and decreased to 2.28 ns in the presence of MP^JSBWMV^:RFP as acceptor (right panel), indicating interaction between GFP:NbPPR and MP^JSBWMV^:RFP. (**d**) Co-IP experiments with NbPPR:GFP or GFP:NbPPR, respectively, and MP^JSBWMV^:RFP co-expressed in *N. benthamiana* leaves revealed interaction of NbPPR with MP^JSBWMV^. IP was conducted with GFP-trap antibodies. Western blots with anti-RFP antibodies were conducted with crude protein extracts to demonstrate that MP^JSBWMV^:RFP and RFP proteins were expressed (Input RFP). Western blots conducted with immunoprecipitated samples and probed with GFP-specific antibodies revealed the presence of NbPPR:GFP and GFP:NbPPR in all samples (Output GFP). When blots conducted with immunoprecipitated samples were probed with anti-RFP antibodies, only bands of MP^JSBWMV^:RFP were detected (Output RFP). Protein sizes were ~85 kDa for NbPPR:GFP and GFP:NbPPR, ~70 kDa for MP^JSBWMV^:RFP and ~27 kDa for RFP.

We next investigated co-localization of GFP:NbPPR and MP^JSBWMV^:RFP. As expected, the proteins co-localized at PD (Fig. 2a). However, when we co-expressed MP^JSBWMV^:RFP together with the chloroplast-localized NbPPR:GFP, no recruitment to PD by MP^JSBWMV^ was observed (Fig. 2b). To confirm the interaction between NbPPR and MP^JSBWMV^, we performed FRET-FLIM experiments. In the presence of MP^JSBWMV^:RFP as acceptor, GFP:NbPPR fluorescence lifetime decreased to 2.28 ns compared to 2.45 ns in the absence of the acceptor, which translates to a FRET efficiency of 6.98% and indicates interaction between the proteins (Fig. 2c, Table 2). Interaction between fluorescent protein-tagged NbPPR and MP^JSBWMV^ was further confirmed in co-IP experiments. GFP:NbPPR precipitated MP^JSBWMV^:RFP, but not free RFP in co-IP experiments using anti-GFP antibodies (Fig. 2d). Interestingly, despite no co-localization was seen in epidermal cells, co-IP experiments also suggested interaction of NbPPR:GFP and MP^JSBWMV^:RFP. Presumably, NbPPR:GFP from chloroplasts broken during grinding with liquid nitrogen found MP^JSBWMV^:RFP during incubation of the GFP-trap beads.

**Table 2.** Fluorescence lifetime values and percentage of FRET are depicted for GFP:NbPPR expressed alone or in the presence of MP^JSBWMV^:RFP Lifetime values in ns; standard deviation (SD) in ns; *N*, number of microscopy images used for analysis; *n*, the number of single measurements used to calculate the average. FRET efficiency was calculated as % FRET. t-test was 3.78E-39 indicating statistical significance.

Proteins	Localization	*N*	*n*	Lifetime [ns]	SD [ns]	% FRET
GFP:NbPPR	Cytoplasm/PD	9	90	2.450	0.062	–
GFP:NbPPR+MP^JSBWMV^:RFP	Cytoplasm/PD	18	218	2.279	0.099	6.97

### NbPPR interacts with RNA

**Fig. 3.**
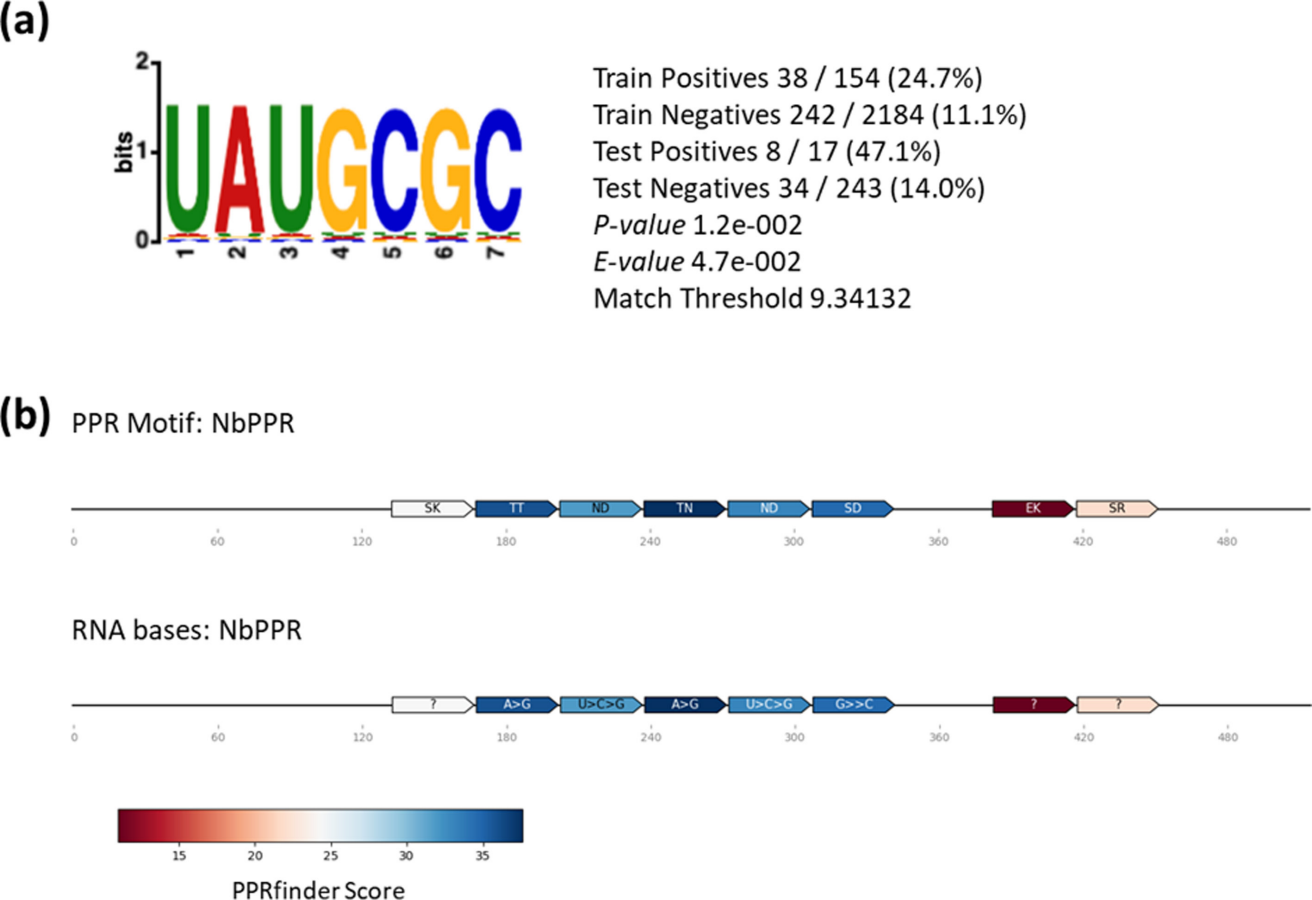
A seven-nucleotide-long RNA-binding motif consistent with the predicted PPR code for NbPPR was found overrepresented among RNAs enriched in GFP:NbPPR RIP. (**a**) One hundred sixty-seven RNAs at least twofold enriched in GFP:NbPPR RIP in comparison to GFP RIP and four RNAs which were highly enriched in GFP:NbPPR RIP but not present in GFP RIP were used to predict the RNA motif with MEME Suite. The motif is shown along with the *P*-value derived from STREME’s binomial test of significance; the *e*-value shows this *P*-value corrected for the number of potential motifs considered. The ‘train positives’ and ‘test positives’ indicate the number of matches found in GFP:NbPPR RIP sequences in training and test datasets, respectively, after removing sites that match previously found motifs. The ‘train negatives’ and ‘test negatives’ report matches found in the training and testing partitions of the control data. ‘Match threshold’ shows the threshold (in bits) for declaring that a given site matches the motif, which is determined by STREME. (**b**) PPR code [57] (https://biolib.com/YaoYinYing/pprcode/, last visited 25 August 2025) was applied to predict the nucleotide target motif of NbPPR. The upper row depicts the predicted RNA-binding amino acids within the identified PPR motifs. The lower row indicates the nucleotides bound preferentially by the amino acid code. The PPRfinder score indicates the probability of binding.

As PPR proteins are RNA-binding proteins [28,29], we investigated which RNAs were bound to the NbPPR when localized to the cytosol and PD using RIP. We speculated that the target RNAs of GFP:NbPPR may provide first insight into the function of the protein during infection. Immunoprecipitated RNAs, which were at least twofold enriched in the GFP:NbPPR samples compared to the GFP samples, were considered as present in the GFP:NbPPR complex (Table 1). RIP identified RNAs encoding proteins with functions in the cytosol and particularly in the cell wall, suggesting that these RNAs are targets of the cytoplasmic/PD-localized GFP:NbPPR. However, many of the identified RNAs were RNAs located in chloroplasts or mitochondria, such as RNAs encoding respiratory chain complex proteins. These RNAs may also represent true targets of the NbPPR protein, which may find these RNAs in the crude extract during RIP. When we searched the 171 RNA sequences enriched in NbGFP:PPR RIP compared to all identified RNAs for the presence of overrepresented nucleotide sequence motifs using MEME, we identified the motif UAUGCGC with statistical significance (Fig. 3a). The identified motif is consistent with the predicted PPR-code for the *N. benthamiana* PPR sequence (Fig. 3b, https://biolib.com/YaoYinYing/pprcode/, last visited 25 August 2025 [57]). Interestingly, we found the identified motif in many plant viral genomes, thus indicating that NbPPR may also directly interact with viral RNA of different plant viruses (Table S5).

### Downregulation of NbPPR affects the number of infection sites formed on leaves

**Fig. 4.**
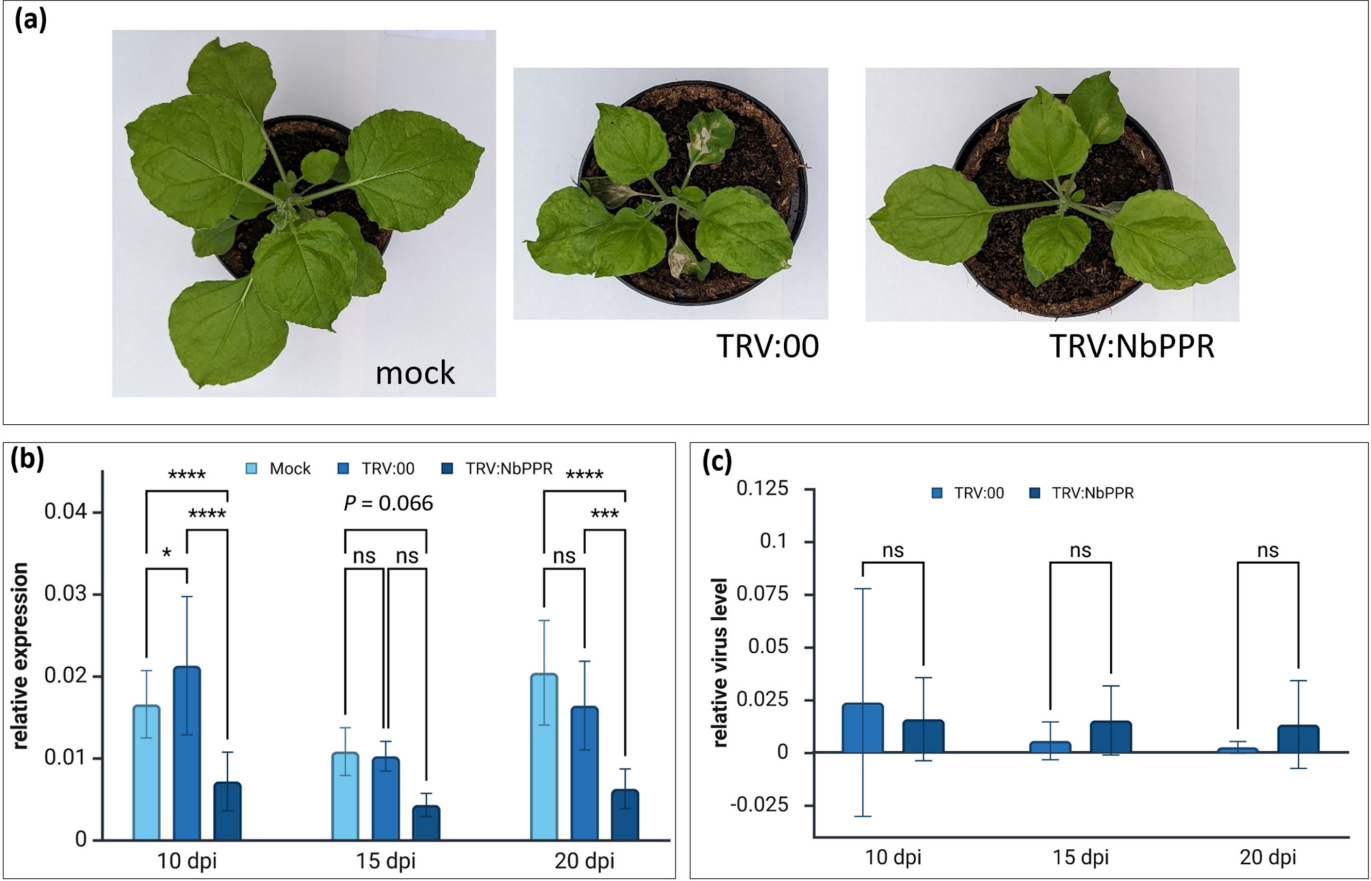
TRV-mediated VIGS of NbPPR in *N. benthamiana*. (**a**) Infection phenotype of *N. benthamiana* plants 10 dpi with TRV:NbPPR and TRV:00. No obvious phenotypic differences were observed between TRV:00 and TRV:NbPPR-inoculated plants, but TRV infection resulted in growth reduction compared to mock-inoculated plants. (**b**) *NbPPR* transcript levels as revealed by quantitative reverse transcription polymerase chain reaction (qRT-PCR) in TRV:NbPPR-infected compared to TRV:00-infected plants and mock controls at 10, 15 and 20 dpi. Relative expression levels are calculated as 2^-ΔCT^. (**c**) TRV RNA1 was quantified by qRT-PCR in TRV:00 and TRV:NbPPR-infected plants. Relative virus level was calculated in relation to the housekeeping genes EF1-alpha and GAPDH. At least three replicates per condition and time point were analysed in three independent experiments. Statistical differences between the sample groups were tested with two-way ANOVA with the Bonferroni multiple comparison test. *P≤0.05, ***P≤0.001 and ****P≤0.0001.

**Fig. 5.**
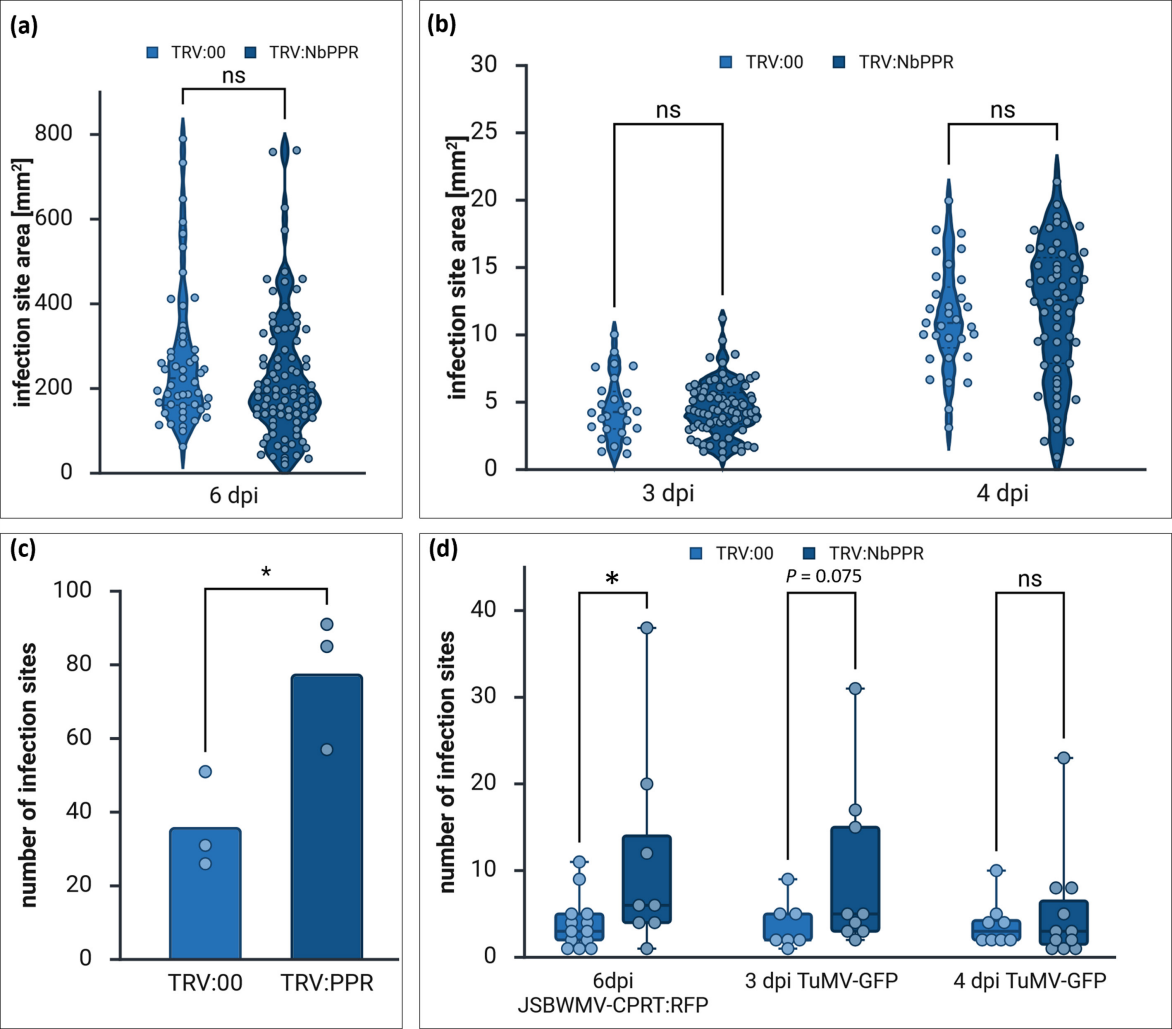
Influence of NbPPR on the size and number of JSBWMV-CPRT:RFP and TuMV-GFP infection sites in *N. benthamiana*. (**a, b**) Area of infection sites formed in leaves silenced for NbPPR expression or in control leaves by (**a**) JSBWMV-CPRT:RFP six dpi or (**b**) TuMV-GFP three and four dpi. Two-way ANOVA with Bonferroni multiple comparison test was applied to investigate statistical significance. Points in the graphs indicate the numbers of individual infection sites measured over all replicates in at least three independent experiments. (**c, d**) Number of fluorescent viral infection sites formed on leaves silenced for NbPPR or control leaves. (**c**) Mean infection site numbers obtained in NbPPR-silenced or control leaves are shown in the bar graph. Welch’s test was used to calculate significant differences. Points in the graph indicate the sum of infection sites per treatment. (**d**) Number of fluorescent viral infection sites per leaf are shown for each virus and time point. Leaves where no infection was obtained were excluded from the analysis. The box plots depict minimum to maximum values, and horizontal lines represent median values. Mann–Whitney U test was applied to calculate statistical differences. Points in the graphs indicate the sum of infection sites per leaf. TRV:NbPPR was used to downregulate the expression of the NbPPR protein in *N. benthamiana*. JSBWMV-CPRT:RFP RNA or TuMV-GFP crude leaf extract were inoculated 10 days after infection with TRV:NbPPR or TRV:00. *P≤0.05; ns, not significant.

To investigate the role of NbPPR during virus infection, we downregulated NbPPR expression using VIGS. Infection of *N. benthamiana* with TRV:NbPPR or TRV:00 caused growth reduction compared to mock-inoculated plants, but no phenotypic changes between TRV:NbPPR and TRV:00 infection were observed (Fig. 4a). After 10, 15 and 20 days of TRV infection, we determined the expression of *NbPPR* in relation to the reference genes GAPDH and EF1alpha using quantitative real-time RT-PCR. We observed a significant downregulation of *NbPPR* in leaves of TRV:NbPPR-infected plants at 10 and 20 dpi (Fig. 4b). No significant differences in the accumulation of TRV RNA in the leaves were observed between the different TRV variants (Fig. 4c). At 10 dpi with TRV:NbPPR or TRV:00 to establish VIGS, plants were infected with JSBWMV-CPRT:RFP or the unrelated potyvirus TuMV-GFP, and fluorescent infection site sizes were quantified. No significant difference in the area of infection sites for JSBWMV-CPRT:RFP (Fig. 5a) and TuMV-GFP (Fig. 5b) was detected. However, substantially more fluorescent infection sites developed on plants silenced for TRV:NbPPR than on control plants (Fig. 5c, d). Thus, downregulation of NbPPR appears to affect plant susceptibility to JSBWMV and TuMV, but not virus movement, once infection is established.

## Discussion

Plant viruses interact with and manipulate their host cells in various ways to achieve successful infection, replication and movement. In this study, we identified an *N. benthamiana* P-type PPR as an MP-interacting protein. The NbPPR localized either to chloroplasts or to the cytosol and PD, depending on the position of the fluorescent protein tag. It is likely that N-terminal tagging of NbPPR with a fluorescent protein tag masked the chloroplast targeting signal, which was predicted in the N-terminal region of the protein. However, the specific localization of NbPPR to PD and cytosol when fused at the N-terminus to fluorescent protein tags suggests that the protein is dually targeted to chloroplasts and cytosol/PD. Consistently, we identified the NbPPR as interaction partner of MP^JSBWMV^, which is not localized to chloroplasts but PD. Close proximity between NbPPR and MP^JSBWMV^ at PD, suggesting interaction of the proteins, was confirmed by FRET-FLIM experiments. Dual targeting of PPR proteins is not uncommon; 10% of the *Arabidopsis* PPR proteins localize to both chloroplast and mitochondrion [66]. Dual targeting of PPR proteins also occurs between chloroplast and nucleus or mitochondrion and nucleus and also cytosolically localized PPR proteins have been described [67,68]. Knowledge on functions of PPR proteins outside chloroplast or mitochondria is scarce. However, it was shown that the PPR-containing *suppressor of the abscisic acid receptor (ABAR) overexpressor* (SOAR1), dually targeted to the nucleus and cytoplasm, acts in the nucleus by negatively regulating the abscisic acid-signalling pathway [67,69]. A function for the cytoplasmic SOAR1 has not yet been demonstrated. The cytosolic rice *CYTOPLASM-LOCALIZED PPR1* (OsCPPR1) downregulates transcription of the *GOLDEN-LIKE1* (OsGLK1) transcription factor by binding to single-stranded regions of *OsGLK1* mRNAs and hence regulates plastid development and maintenance [68].

The specificity of RNA binding by PPRs results from the modular structure of the PPR motifs and the interactions between specific amino acids in the PPR turns with target nucleotides in RNA [28,57, 70, 71]. Different models have been developed to predict PPR target RNA motifs. To experimentally derive a target motif for the MP-interacting NbPPR, we here used RNA sequences overrepresented in RIP experiments using overexpressed PD/cytosol-localized NbPPR in plant extracts. The motif identified using the RNAs specifically immunoprecipitated with PD/cytosol-localized NbPPR is consistent with the predicted motif using the algorithm provided by the PPR code tool [57] (https://biolib.com/YaoYinYing/pprcode/, last visited 25 August 2025) and the *N. benthamiana* PPR protein sequence as input. Thus, the sequence we identified using our NbPPR-bound RNAs likely recognizes specific targets of the MP-interacting NbPPR. Whether the NbPPR target RNAs have specific functions with respect to PD and or systemic RNA transport remains to be investigated.

The majority of RNA targets of the NbPPR protein identified here are organellar transcripts encoding subunits of the NADH dehydrogenase complex I (NADH-quinone oxidoreductase), involved in electron shuttling during the electron transport chain in mitochondria and cyclic electron transport in chloroplasts. Plastids and mitochondria are the main sources for ROS and defence signalling [72,74]. In addition, important steps in the synthesis of phytohormone precursors like salicylic acid, jasmonic acid, abscisic acid or ethylene take place in the chloroplast [75,76].

Interestingly, PPR activity in editing and splicing of *NADH dehydrogenase* transcripts has been shown to play a role in modulating cellular redox state and to alter disease susceptibility [77,79]. The *Arabidopsis* P-type PPR protein RTP7, for instance, was shown to modulate mitochondrial ROS levels via its role in splicing of *nad7*, which encodes a subunit of mitochondrial NADH dehydrogenase [79]. *rtp7* plants exhibited higher resistance to *Phytophthora parasitica* due to an elevated burst of mitochondrial ROS. In rice, a mitochondrion-localized PPR protein involved in splicing of *NADH dehydrogenase complex I* transcripts was shown to play a role in resistance against fungal pathogens, and mutants had elevated ROS levels [78]. Also chloroplastic NADH dehydrogenase-like complex components are targeted by PPR proteins. The *Arabidopsis thaliana* OCP3 protein, co-expressed with PPR proteins as well as different chloroplastic PPR proteins involved in editing chloroplastic NADH dehydrogenase*–*like complex components, was shown to play a role in resistance against fungal pathogens [77]. Thus, the modulation of cyclic electron flow around photosystem I by PPR proteins and upon biotic stress appears to play a crucial role in conferring resistance. Hence, control of mitochondrial and plastid redox state appears to control plant immunity.

We observed enhanced susceptibility towards virus infection upon downregulation of the MP^JSBWMV^-interacting NbPPR in *N. benthamiana* using VIGS, as more fluorescent infection sites were formed on inoculated leaves. As we identified NADH dehydrogenase complex components as targets for NbPPR, it appears probable that NbPPR functions in modulating electron transport and in consequence cellular redox state. Remarkably, defects in mitochondrial NADH dehydrogenase complex I function have been shown to be accompanied by modulated responses to TMV (species *Tobamovirus tabaci*) infection [80]. TMV infection of tobacco plants carrying the N resistance gene resulted in fewer and smaller infection sites in plants with impaired complex I function compared to the wild-type. In these plants, the expression and activity of several antioxidant enzymes were modified, suggesting extensive antioxidant crosstalk and acclimation between the mitochondria and other organelles to maintain whole cell redox balance. Also, the chloroplastic NADH dehydrogenase-like complex has been shown to be involved in regulating responses to virus infection. TuMV infection first upregulated and then downregulated the *NADH dehydrogenase complex I subunit M* gene expression, and silencing of this gene enhanced susceptibility to infection, while overexpression increased resistance [81]. Nevertheless, given a postulated function of NbPPR in the cytosol and/or at PD, we would expect also cytosolic and/or PD/cell wall-localized RNA targets for GFP:NbPPR. Indeed, the list of identified NbPPR targets includes several RNAs that encode proteins and enzymes whose activity leads to altered cell wall structures, i.e. a chitinase, 4-coumarate:CoA ligase-like 7 and germin-like protein.

**Fig. 6.**
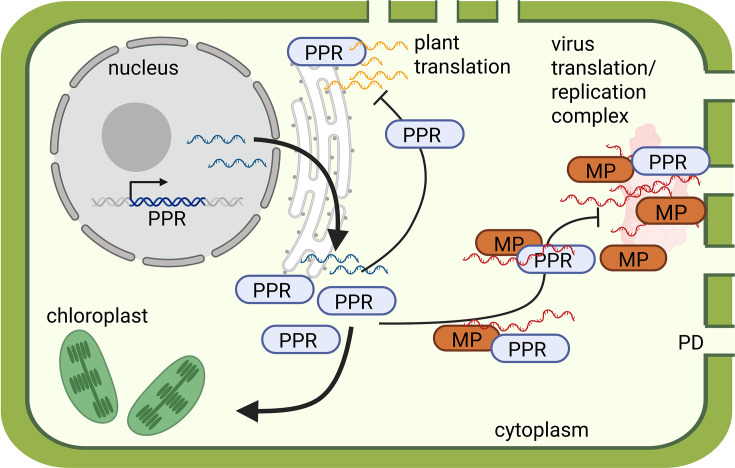
Model for NbPPR function during virus infection NbPPR is encoded in the nucleus and translated in the cytoplasm at the ER. NbPPR is transported to the chloroplast via its N-terminal signal peptide. JSBWMV infection starts with initial translation and replication to establish infection. NbPPR can interact with the viral MP and may act on viral RNA (dark orange) as a target through recognition of the PPR-code motif UAUGCGC present in viral RNA. Interaction between MP^JSBWMV^ and NbPPR occurs in the cytoplasm and at PD. The interaction between the NbPPR and the viral components modulates virus replication or translation. The effect of MP^JSBWMV^–NbPPR interaction in the cytoplasm/at PD may also be more indirect: RNA targets (light orange) of NbPPR in the cytoplasm or targets in the chloroplasts may be changed or deregulated, because of fewer NbPPR in the chloroplast. As we identified NADH-dehydrogenase components as NbPPR targets, modulated NADH-dehydrogenase activity may affect plastid and cellular redox status, possibly resulting in a defence state of the plant cell and less infection. Image created in BioRender. Strauch, C. (2026) https://BioRender.com/myq2k9k.

To put our results into context with existing literature and to stimulate further research on PPR functions in plant virus infection, we here propose a model for the role of NbPPR during JSBWMV infection (Fig. 6). Modulations of the plastidial redox state by PPR proteins can lead to altered PD transport [82,83]. Knockdown of the PPR protein IPI1 in *N. benthamiana* resulted in decreased PD density and decreased intercellular trafficking [84]. Thus, by interacting with PPRs, viral proteins may interfere with PPR function to modulate steps in the virus infection cycle (Fig. 6). By binding to NbPPR in the cytoplasm, MP^JSBWMV^ may keep NbPPR from acting on its targets. Interference with chloroplastic NbPPR functions by MP may affect electron transport and redox state, which may result in enhanced susceptibility to infection. Interference of MP^JSBWMV^ with NbPPR function in the chloroplast may also affect retrograde signalling and PD transport. However, also a more direct role of MP interacting with NbPPR is thinkable. By binding NbPPR in the cytoplasm, MP^JSBWMV^ may change PPR localization towards other subcellular components and RNA targets may be altered. Cytidine-to-uridine (C-to-U) RNA editing by PPR proteins, which usually takes place in organelles, has been shown to occur in the cytoplasm upon overexpression of *Physcomitrium patens* PPR56, PPR65 and PPR78 proteins [85]. Interestingly, over 900 off-targets were affected by PPR overexpression in the cytoplasm. By binding to and interfering with NbPPR function in the cytoplasm/at PD, MP^JSBWMV^ could directly influence cell wall structure and may modulate PD control. This is particularly interesting with respect to the identified putative target RNAs encoding cell wall proteins. However, our infection experiments did not resolve significant differences in infection site size. Finally, MP^JSBWMV^ may also directly interact with NbPPR to recruit it to the viral RNA as NbPPR target. Interestingly, many plant virus RNA genomes encode the identified NbPPR motif in different viral ORFs and silencing of NbPPR positively affected the development of infection sites also of the JSBWMV unrelated TuMV (Fig. 5 d, *P*-value 0.073). Thus, it is tempting to speculate that by binding to viral RNA, virus RNA stability, replication or translation may be regulated during the infection cycle.

## Conclusion

We here identified the chloroplastic/cytosolic NbPPR protein as host interaction partner of MP^JSBWMV^ and showed that plants with reduced NbPPR expression are more susceptible to virus infection. We also obtained evidence that NbPPR functions in the chloroplast by targeting chloroplastic NADH-dehydrogenase complex subunits and that it may function in the cytosol and target RNAs involved in cell wall metabolism. Whether NbPPR has anti-viral and possibly also pro-viral functions during infection and which role it may play in regard to possible viral RNA targets requires further investigation. Taken together, we here identify a novel host regulator of virus infection in plants. Given that PPR proteins are increasingly engineered for biotechnological applications [30,86], PPRs may also be developed into tools to control virus infection in the future.

## Supplementary material

10.1099/jgv.0.002260 Supplementary Material 1.

## Abbreviations

CP: capsid protein
CPRT: capsid protein read-through
CWMV: Chinese wheat mosaic virus
dpi: days post-infection
FLIM: fluorescence lifetime imaging microscopy
FRET: fluorescence resonance energy transfer
IP: immunoprecipitation
JSBWMV: Japanese soil-borne wheat mosaic virus
MP: movement protein
NEB: New England Biolabs
PD: plasmodesmata
PM: plasma membrane
RIP: RNA immunoprecipitation
TMV: tobacco mosaic virus
TRV: tobacco rattle virus
TuMV: turnip mosaic potyvirus
UBA2C: UBP1-associated protein 2C
VIGS: virus-induced gene silencing
