# Supplementary material for: A P-type pentatricopeptide repeat-containing protein interacts with the Japanese soil-borne wheat mosaic virus movement protein and modulates susceptibility to infection
Source: J Gen Virol. 2026 Jun 4;107(6):002260. doi: 10.1099/jgv.0.002260 (PMC13235989; doi:10.1099/jgv.0.002260)
Supplement: Supplementary Material 1. [file jgv-107-02260-s001.pdf]

Supplementary material

A P-type pentatricopeptide-repeat protein interacts with the Japanese soil-borne wheat mosaic virus movement protein and modulates susceptibility to infection

Authors: Claudia J. Strauch[1], Khalid Amari[2], Steffen Ostendorp[3], Ruth Veevers[4], Emmanuel Boutant[5,6], Nico Sprotte[1], Richard J. Morris[4], Julia Kehr[3], Annette Niehl[1]

[1]Julius Kühn Institute (JKI) - Federal Research Centre for Cultivated Plants, Institute for Epidemiology and Pathogen Diagnostics, Messeweg 11-12. 38104 Brunswick, Germany
[2]Julius Kühn Institute (JKI) - Federal Research Centre for Cultivated Plants, Institute for Biosafety in Plant Biotechnology, Erwin-Baur-Str. 27, 06484 Quedlinburg, Germany
[3]Institute for Plant Science and Microbiology, Universität Hamburg, Ohnhorststr. 18, 22609 Hamburg, Germany
[4] Computational and Systems Biology, John Innes Centre, Norwich, UK
[5]Laboratory of Bioimaging and Pathologies, CNRS UMR 7021, Faculty of Pharmacy, University of Strasbourg, 74 Route du Rhin - CS 60024, F-67400, Illkirch, Strasbourg, France
[6]Present address: Biotechnology and Cell Signaling, CNRS UMR7242. ESBS, University of Strasbourg, Bld Sébastien Brant, F-67412, Illkirch, Strasbourg, France

Corresponding author: annette.niehl@julius-kuehn.de

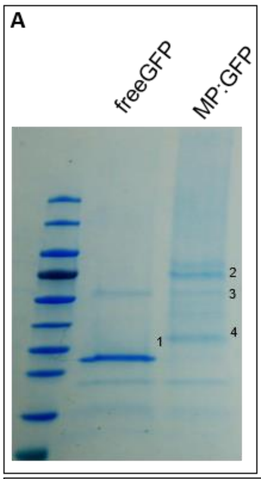

B

Lane No.	Mwobs [kDa]	Identified protein	MW [kDa]	Accesion no.	MAS-COT score	Sequence coverage [%]
1	30	eGFP	32	AZQ25072.1	270	61/83
2	70	37-kDa MP:eGFP	65	YP_009508079.1 JSBWMV AEA29852.1		29/83
3-1	57	ribulose-1,5-bisphosphate carboxylase/oxygenase large subunit	53	P48709.1 Nicotiana debneyi	178	27/87
3-2	57	pentatricopeptide repeat-containing protein At1g62910-like	56	XP_009608994.1 N. tomentosiformis	110	19/91
4	36	MP:eGFP, partial	65	AAS45194.1	123	32/83

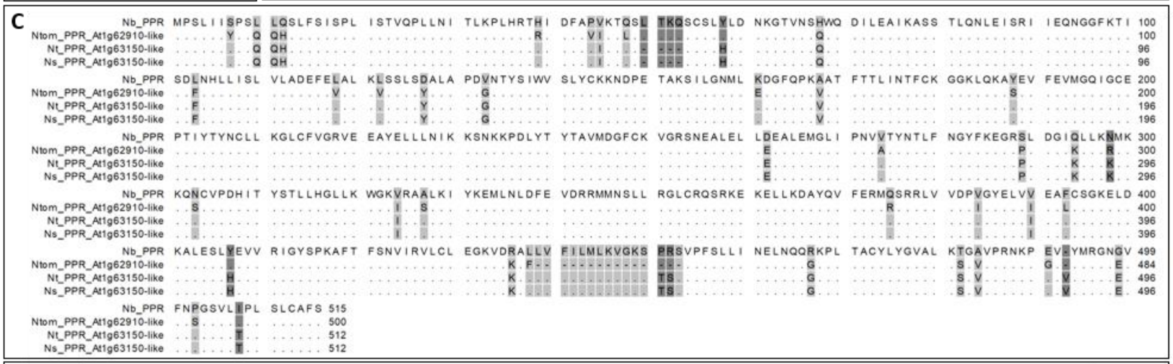

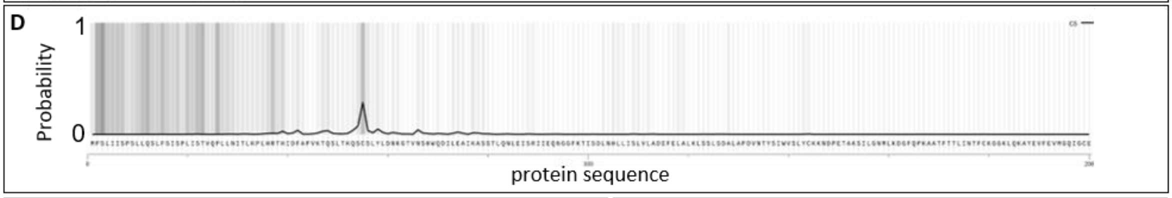

E

Protein type	likelihood
Other	0.2047
Secretory pathway peptide	0.1617
Mitochondrial transfer peptide	0.053
Chloroplast transfer peptide	0.5525
Thylakoid luminal transfer peptide	0.0282

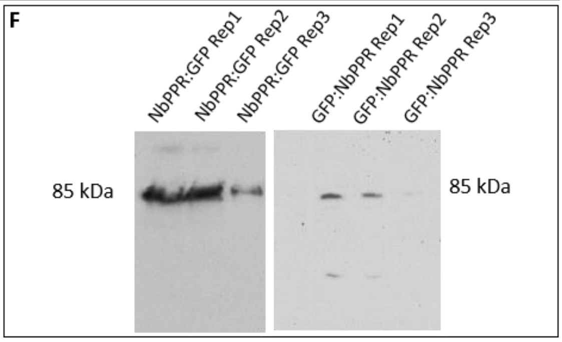

Figure S1. MP^{JSBWMV} precipitates a NbPPR-protein from *N. benthamiana*

(**A**) Colloidal coomassie-stained SDS-PAGE gel showing immunoprecipitated proteins identified in mass spectrometry. Proteins obtained in co-IP experiments after overexpression of MP^{JBSWMV}:GFP or GFP were separated with SDS-PAGE and stained with colloidal coomassie, bands marked with a number were cut out, digested and used in mass spectrometry. (**B**) Table lists the identified proteins, their molecular weight, the MASCOT-score and sequence coverage. (**C**) Alignment of NbPPR amino acid sequences from *N. benthamiana* sharing 89.0 % identity with *N. tomentosiformis*, 94.2 % identity with *N. tabacum* and *N. sylvestris*. (**D**) TargetP2.0 (https://services.healthtech.dtu.dk/service.php?TargetP-2.0 (retrieval date 08.03.2022 (Almagro Armenteros et al. 2019)) suggests the location of the signal-peptide in the protein sequence of NbPPR in the N-terminal 100 amino acids of the protein. (**E**) TargetP2.0 predicted likelihoods for the distribution of NbPPR, with the highest likelihood for a chloroplast-distribution. (**F**) Western blots showing NbPPR:GFP and GFP:NbPPR proteins at approximately 85 kDa using specific antibody against GFP (Rep, replicate).

Table S1. Primer sequences

Primer name	Sequence 5´-3´	Description	Reference
GW_N.b_PRP_For1	GGGGACAAGTTTGTACAAAAAAGCAGGC TTA AAC ATGCCTTCTTTAATTATCTC	Preparation for BP-reaction (GATEWAY): add attB-sites to PPR protein (C- and N-terminal fusion in frame with Kozak-element)	
GW_N.b_PRP_Rev1	GGG GAC CAC TTT GTA CAA GAA AGC TGG GTC TGAAAAAGCACACAATG	Preparation for BP-reaction (GATEWAY): add attB-sites to PPR protein (C-terminal fusion without stop-codon)	
GW_N.b_PRP-Stop_Rev2	GGG GAC CAC TTT GTA CAA GAA AGC TGG GTC TCA TGAAAAAGCACACAATG	Preparation for BP-reaction (GATEWAY): add attB-sites to PPR protein (N-terminal fusion)	
VIGS_Nb_PPR_For2	CTAGTCTAGAAAGGCATTCACTTTCTCTAATG	Amplification of PPR insert introducing restriction sites for Xba1 for cloning VIGS TRV-RNA2:PPR	
VIGS_Nb-myPPR-CS-Rev-2	CGGGGTACCTCATGAAAAAGCACACAATGAC	Amplification of PPR insert introducing restriction sites for Kpn1 for cloning VIGS TRV-RNA2:PPR	
qPCR_Nt_GAPDH_For1	GTGCCAAGAAGGTTGTGATC	Housekeeping gene glyceraldehyde-3-phosphate dehydrogenase as reference for qPCR for *N. benthamiana*	
qPCR_Nt_GAPDH_Rev1	CAAGGCAGTTGGTAGTGCAA	Housekeeping gene glyceraldehyde-3-phosphate dehydrogenase as reference for qPCR for *N. benthamiana*	
qPCR_Nb_EF1-alpha_For1	GTAGGTCCAAAGGTCACAACCAT	Housekeeping gene elongation factor 1-alpha as reference for qPCR for *N. benthamiana*	
qPCR_Nb_EF1-alpha_Rev1	GTACTGTCCCTGTTGGTCGT	Housekeeping gene elongation factor 1-alpha as reference for qPCR for *N. benthamiana*	
qPCR_Nb_PPR_For2	GAGGTGGATAGAAGGATG	Primer for PPR protein for measuring gene expression in	

		qPCR for *N. benthamiana*	
qPCR_Nb_PPR_Rev2	ACCCAACAGGATCAACAA	Primer for PPR protein for measuring gene expression in qPCR for *N. benthamiana*	
qPCR_TRV-RNA1_For1	GACGTGTGTACTCAAGGGTT	Determination of TRV accumulation in qPCR by detection of TRV RNA1	Published in Boutsika et al., 2004 (PpK20 RNA-1 6113-6132)
qPCR_TRV-RNA1_Rev1	GGGCGTAATAACGCTTACG	Determination of TRV accumulation in qPCR by detection of TRV RNA1	Published in Boutsika et al., 2004 (3' terminus of all tobravirus RNAs)
CPRT_RFP _For1	TGGCAGCTGAGAACCTAGAG	Amplification of CP-RT:RFP from pH7-CP-RT:GFP	
Bgl2_RFP_Rev1	GAAGATCTTTAGGCGCCGGTGG	Addition of BglII restriction sites to RFP 3' end from pH7-CP-RT:RFP	

Table S2. RNAs enriched in GFP:NbPPR RIP used for RNA binding motif search

Annotation: *N. benthamiana* draft genome	log2 fold change	number of reads in GFP: NbPPR RIP	Scaf-fold length	GFP				GFP:NbPPR				Annotation
				Rep1		Rep2		Rep1		Rep2		
				Reads	Counts per million	Reads	Counts per million	Reads	Counts per million	Reads	Counts per million	
Niben101Scf04451g00027.1	2.07	1973	246	155	1.92	434	3.51	1005	12.06	968	9.84	Mitochondr+T4:T161ial protein, putative [Medicago truncatula]
Niben101Scf05689g02012.1	2.62	2480	429	122	1.51	406	3.29	1260	15.12	1220	12.40	NADH-quinone oxidoreductase subunit H
Niben101Scf15885g00035.1	2.59	2667	852	120	1.49	487	3.94	1504	18.04	1163	11.82	conserved hypothetical protein [Ricinus communis] gb|EEF25773.1| conserved hypothetical protein [Ricinus communis]
Niben101Scf01681g02009.1	2.22	986	3393	110	1.37	119	0.96	584	7.01	402	4.09	Protein IQ-DOMAIN 14
Niben101Scf08162g00010.1	2.81	2044	300	93	1.15	276	2.23	1072	12.86	972	9.88	NADH-quinone oxidoreductase subunit H 1
Niben101Scf04432g06003.1	2.04	1426	345	91	1.13	398	3.22	793	9.51	633	6.43	conserved hypothetical protein [Ricinus communis] gb|EEF45986.1| conserved hypothetical protein [Ricinus communis]
Niben101Scf11347g00005.1	2.31	1311	879	82	1.02	259	2.10	675	8.10	636	6.47	NADH-quinone oxidoreductase subunit H
Niben101Scf04048g00015.1	2.58	1598	687	81	1.01	263	2.13	908	10.89	690	7.01	orf109 (mitochondrion) [Panax ginseng] gb|AHJ81034.1| orf109 (mitochondrion) [Panax ginseng]
Niben101Scf02309g03017.1	2.31	1350	378	73	0.91	305	2.47	714	8.57	636	6.47	conserved hypothetical protein [Ricinus communis] gb|EEF25773.1| conserved

											hypothetical protein [Ricinus communis] conserved hypothetical protein [Ricinus communis] gb|EEF45986.1| conserved hypothetical protein [Ricinus communis]
Niben101Scf05540g00010.1	2.18	1005	345	56	0.70	262	2.12	569	6.83	436	4.43	
Niben101Scf00854g06020.1	2.17	730	887	52	0.65	152	1.23	330	3.96	400	4.07	Unknown protein
Niben101Scf01184g09019.1	2.01	476	3270	45	0.56	93	0.75	259	3.11	217	2.21	hero resistance protein 3 homologue [Solanum lycopersicum]
Niben101Scf02074g06077.1	2.12	296	570	41	0.51	34	0.28	158	1.90	138	1.40	NADH dehydrogenase subunit 2 (mitochondrion) [Isoetes engelmannii]
Niben101Scf00367g07004.1	2.92	877	648	33	0.41	118	0.96	519	6.23	358	3.64	orf109 (mitochondrion) [Panax ginseng] gb|AHJ81034.1| orf109 (mitochondrion) [Panax ginseng]
Niben101Scf02074g01039.1	2.29	326	303	33	0.41	38	0.31	204	2.45	122	1.24	Ribosomal protein S10 [Medicago truncatula]
Niben101Scf02290g05024.1	2.30	97	2381	28	0.35	4	0.03	59	0.71	38	0.39	OBP3-responsive gene 1 LENGTH=670
Niben101Scf03515g00078.1	2.25	309	1333	27	0.34	47	0.38	143	1.72	166	1.69	NADH-quinone oxidoreductase subunit D
Niben101Scf00916g01010.1	2.04	188	6666	26	0.32	22	0.18	124	1.49	64	0.65	Unknown protein
Niben101Scf00482g08002.1	2.36	496	204	25	0.31	111	0.90	277	3.32	219	2.23	Unknown protein
Niben101Scf14427g00008.1	2.08	272	1044	24	0.30	52	0.42	143	1.72	129	1.31	DNA polymerase (mitochondrion) [Silene vulgaris]
Niben101Scf06928g00013.1	3.13	687	279	24	0.30	75	0.61	410	4.92	277	2.82	orf109 (mitochondrion) [Panax ginseng] gb|AHJ81034.1| orf109 (mitochondrion) [Panax ginseng]
Niben101Scf05250g00010.1	2.18	207	7708	20	0.25	27	0.22	143	1.72	64	0.65	Unknown protein
Niben101Scf03515g00058.1	2.35	308	339	19	0.24	58	0.47	140	1.68	168	1.71	BnaUnng03880D, partial [Brassica napus]
Niben101Scf02041g00002.1	2.23	204	1315	18	0.22	32	0.26	101	1.21	103	1.05	Chitinase 8

Niben101Scf00849g01019.1	2.08	228	414	18	0.22	49	0.40	108	1.30	120	1.22	NADH-ubiquinone oxidoreductase chain 4
Niben101Scf03431g00004.1	2.02	215	453	18	0.22	47	0.38	97	1.16	118	1.20	NADH-quinone oxidoreductase subunit H
Niben101Scf18129g00003.1	2.38	183	1125	17	0.21	22	0.18	88	1.06	95	0.97	NADH-quinone oxidoreductase subunit D
Niben101Scf09696g01037.1	2.22	241	420	16	0.20	51	0.41	118	1.42	123	1.25	conserved hypothetical protein [Ricinus communis] gb|EEF25773.1| conserved hypothetical protein [Ricinus communis]
Niben101Scf08792g00008.1	2.06	43	300	16	0.20	2	0.02	19	0.23	24	0.24	Protein Ycf2
Niben101Scf10881g00025.1	2.20	248	660	16	0.20	54	0.44	145	1.74	103	1.05	NADH-ubiquinone oxidoreductase chain, putative [Ricinus communis] gb|EEF27701.1| NADH-ubiquinone oxidoreductase chain, putative [Ricinus communis]
Niben101Scf14482g00006.1	2.12	119	285	14	0.17	16	0.13	67	0.80	52	0.53	BnaCnng48510D [Brassica napus]
Niben101Scf08015g02002.1	2.10	157	249	12	0.15	34	0.28	78	0.94	79	0.80	cytochrome c biogenesis FN (mitochondrion) [Hevea brasiliensis]
Niben101Scf05439g02038.1	2.41	125	378	12	0.15	12	0.10	86	1.03	39	0.40	orf116d (mitochondrion) [Batis maritima] gb|AIC83424.1| orf116d (mitochondrion) [Batis maritima]
Niben101Scf09825g01007.1	2.00	86	5106	12	0.15	11	0.09	53	0.64	33	0.34	Unknown protein
Niben101Scf04114g01046.1	2.07	141	885	11	0.14	31	0.25	72	0.86	69	0.70	NADH dehydrogenase subunit 5 (mitochondrion) [Silene vulgaris]
Niben101Scf18129g00016.1	3.11	255	381	11	0.14	24	0.19	136	1.63	119	1.21	BnaCnng48510D [Brassica napus]
Niben101Scf03431g00012.1	2.12	194	306	11	0.14	54	0.44	107	1.28	87	0.88	4-coumarate--CoA ligase-like 7 [Morus notabilis] gb|EXC31783.1| 4-coumarate--CoA ligase-like 7 [Morus notabilis]

Niben101Scf00853g03001.1	2.10	152	213	11	0.14	33	0.27	93	1.12	59	0.60	orf34 gene product (mitochondrion) [Daucus carota subsp. sativus] gb|AEY81172.1| orf34 (mitochondrion) [Daucus carota subsp. sativus]
Niben101Scf06884g02004.1	2.48	76	807	10	0.12	5	0.04	25	0.30	51	0.52	Sec23/Sec24 protein transport family protein LENGTH=880
Niben101Scf00236g01012.1	2.02	66	225	10	0.12	8	0.06	30	0.36	36	0.37	Unknown protein
Niben101Scf02749g04008.1	2.32	178	627	10	0.12	38	0.31	97	1.16	81	0.82	Germin-like protein subfamily 3 member 3
Niben101Scf00466g04001.1	2.08	80	531	9	0.11	12	0.10	43	0.52	37	0.38	Cytochrome b
Niben101Scf05519g01010.1	2.43	93	345	9	0.11	10	0.08	46	0.55	47	0.48	Unknown protein
Niben101Scf01374g02004.1	2.76	214	693	8	0.10	37	0.30	124	1.49	90	0.91	Cytochrome C assembly protein LENGTH=256
Niben101Scf01745g07016.1	2.86	230	693	8	0.10	37	0.30	134	1.61	96	0.98	Cytochrome C assembly protein LENGTH=256
Niben101Scf02497g07001.1	2.53	157	693	8	0.10	27	0.22	93	1.12	64	0.65	Cytochrome C assembly protein LENGTH=256
Niben101Scf00485g00013.1	2.67	190	693	8	0.10	33	0.27	108	1.30	82	0.83	Cytochrome C assembly protein LENGTH=256
Niben101Scf06928g00002.1	2.25	60	327	8	0.10	6	0.05	33	0.40	27	0.27	PSII 32 kDa protein [Vitis vinifera] emb|CAQ77603.1| PSII 32 kDa protein [Vitis vinifera] gb|ACS15235.1| PSII 32 kDa protein [Vitis vinifera]
Niben101Scf05654g00012.1	2.30	88	519	8	0.10	12	0.10	42	0.50	46	0.47	Unknown protein
Niben101Scf04784g03004.1	2.48	42	3221	7	0.09	2	0.02	30	0.36	12	0.12	F-box protein
Niben101Scf05340g00009.1	2.06	33	648	7	0.09	2	0.02	8	0.10	25	0.25	product [Oryza sativa Japonica Group]
Niben101Scf22627g00001.1	2.12	54	660	6	0.07	6	0.05	14	0.17	40	0.41	30S ribosomal protein S2
Niben101Scf03924g03008.1	2.53	65	1608	6	0.07	6	0.05	24	0.29	41	0.42	RNA-directed DNA polymerase [Thermoanaerobacter ethanolicus] gb|EGD52597.1| RNA-directed DNA polymerase (Reverse transcriptase)[Thermoanaerobacter ethanolicus JW 200]
Niben101Scf00107g03008.1	2.90	153	771	6	0.07	19	0.15	102	1.22	51	0.52	Cysteine-rich venom protein

Niben101Scf02929g03029.1	2.05	48	228	6	0.07	6	0.05	16	0.19	32	0.33	Unknown protein
Niben101Scf02929g04040.1	2.17	93	558	6	0.07	19	0.15	30	0.36	63	0.64	ATP synthase protein MI25
Niben101Scf03515g00073.1	2.97	86	1272	6	0.07	6	0.05	49	0.59	37	0.38	NADH-quinone oxidoreductase subunit D
Niben101Scf15885g00031.1	2.41	109	516	6	0.07	21	0.17	59	0.71	50	0.51	NADH-quinone oxidoreductase subunit D
Niben101Scf02002g01024.1	3.03	224	693	6	0.07	37	0.30	130	1.56	94	0.96	Cytochrome C assembly protein LENGTH=256
Niben101Scf03012g03019.1	2.24	82	1471	6	0.07	12	0.10	60	0.72	22	0.22	RING finger protein 150
Niben101Scf03267g00012.1	2.20	45	284	6	0.07	4	0.03	13	0.16	32	0.33	Unknown protein
Niben101Scf00199g03003.1	2.56	37	4330	6	0.07	2	0.02	18	0.22	19	0.19	nucleotide binding protein, putative [Ricinus communis] gb|EEF34442.1| nucleotide binding protein, putative [Ricinus communis]
Niben101Scf00914g03002.1	2.44	152	195	6	0.07	39	0.32	86	1.03	66	0.67	30S ribosomal protein S3
Niben101Scf05776g01029.1	2.69	48	1011	5	0.06	3	0.02	16	0.19	32	0.33	Protein Ycf2
Niben101Scf04883g00005.1	2.02	33	336	5	0.06	4	0.03	16	0.19	17	0.17	Unknown protein
Niben101Scf02309g03024.1	2.01	26	357	5	0.06	2	0.02	7	0.08	19	0.19	Cytochrome b
Niben101Scf06058g00015.1	2.15	23	2123	4	0.05	2	0.02	13	0.16	10	0.10	Pimeloyl-[acyl-carrier protein] methyl ester esterase
Niben101Scf04114g01045.1	2.31	45	828	4	0.05	6	0.05	27	0.32	18	0.18	60S ribosomal protein L2, mitochondrial
Niben101Scf04114g01042.1	2.02	31	1116	4	0.05	2	0.02	4	0.05	27	0.27	NADH-quinone oxidoreductase subunit D
Niben101Scf09075g03002.1	2.43	28	1221	4	0.05	2	0.02	12	0.14	16	0.16	xyloglucan endotransglucosylase/hydrolase 6 LENGTH=292
Niben101Scf00823g01010.1	2.13	23	3629	4	0.05	2	0.02	14	0.17	9	0.09	Protein TRANSPORT INHIBITOR RESPONSE 1
Niben101Scf04953g06007.1	2.55	60	1447	4	0.05	8	0.06	30	0.36	30	0.30	Syntaxin-71
Niben101Scf12745g00004.1	2.04	22	276	4	0.05	2	0.02	8	0.10	14	0.14	transport membrane protein, partial (mitochondrion) [Butomus umbellatus]
Niben101Scf01188g05004.1	2.54	30	4239	4	0.05	2	0.02	14	0.17	16	0.16	Tetratricopeptide repeat (TPR)-like superfamily protein LENGTH=977

Niben101Scf01795g04024.1	2.00	45	1356	4	0.05	9	0.07	28	0.34	17	0.17	Glutamyl-tRNA(Gln) amidotransferase subunit A
Niben101Scf04708g00011.1	2.31	72	6240	4	0.05	16	0.13	34	0.41	38	0.39	cellulose synthase 6 LENGTH=1084
Niben101Scf05392g01022.1	2.09	16	690	4	0.05	1	0.01	6	0.07	10	0.10	Peroxiredoxin-2B
Niben101Scf02074g06084.1	2.36	70	366	4	0.05	14	0.11	32	0.38	38	0.39	NADH dehydrogenase subunit 5 (mitochondrion) [Silene vulgaris]
Niben101Scf02816g12003.1	2.07	45	207	4	0.05	8	0.06	29	0.35	16	0.16	ATP-dependent Clp protease proteolytic subunit
Niben101Scf02816g16025.1	2.37	42	6323	4	0.05	5	0.04	20	0.24	22	0.22	brefeldin A-inhibited guanine nucleotide-exchange protein [Medicago truncatula]
Niben101Scf13429g03011.1	2.41	61	599	4	0.05	10	0.08	29	0.35	32	0.33	Bifunctional inhibitor/lipid-transfer protein/seed storage 2S albumin superfamily protein LENGTH=134
Niben101Scf00150g00007.1	2.06	52	1814	4	0.05	10	0.08	36	0.43	16	0.16	Tubulin alpha chain
Niben101Scf03460g05002.1	2.39	27	3940	4	0.05	2	0.02	13	0.16	14	0.14	Formate--tetrahydrofolate ligase
Niben101Scf01616g01007.1	2.65	23	363	4	0.05	1	0.01	10	0.12	13	0.13	Ribosomal protein S3, mitochondrial
Niben101Scf14482g00003.1	3.06	137	459	4	0.05	20	0.16	80	0.96	57	0.58	orf109 (mitochondrion) [Panax ginseng] gb|AHJ81034.1| orf109 (mitochondrion) [Panax ginseng]
Niben101Scf34790g00004.1	2.07	30	2530	4	0.05	4	0.03	12	0.14	18	0.18	nuclear RNA binding protein A [Spinacia oleracea]
Niben101Scf00883g05009.1	2.20	27	4080	4	0.05	2	0.02	7	0.08	20	0.20	ATP binding microtubule motor family protein LENGTH=1004
Niben101Scf00401g00010.1	2.22	18	1497	4	0.05	1	0.01	6	0.07	12	0.12	Sorting nexin 2B
Niben101Scf05682g00011.1	2.28	38	1886	4	0.05	4	0.03	26	0.31	12	0.12	Alkyl hydroperoxide reductase subunit C
Niben101Scf02474g01021.1	2.22	24	3188	4	0.05	2	0.02	12	0.14	12	0.12	Pyrophosphate-energized vacuolar membrane proton pump 1
Niben101Scf00594g02002.1	2.03	47	1609	4	0.05	8	0.06	13	0.16	34	0.35	Asparagine synthetase [glutamine-hydrolyzing] 2

Niben101Scf02798g00047.1	2.17	53	228	3	0.04	14	0.11	28	0.34	25	0.25	Unknown protein
Niben101Scf00108g06007.1	2.01	26	1179	3	0.04	4	0.03	16	0.19	10	0.10	stomatal cytokinesis defective / SCD1 protein (SCD1) LENGTH=1187
Niben101Scf01983g07005.1	2.40	62	333	3	0.04	14	0.11	30	0.36	32	0.33	Unknown protein
Niben101Scf03723g00009.1	2.29	31	1383	3	0.04	4	0.03	17	0.20	14	0.14	MATE efflux family protein LENGTH=506
Niben101Scf00949g03003.1	2.72	26	2673	2	0.02	2	0.02	8	0.10	18	0.18	MORN (Membrane Occupation and Recognition Nexus) repeat-containing protein LENGTH=871
Niben101Scf00451g00027.1	2.67	50	345	2	0.02	9	0.07	20	0.24	30	0.30	orf105 (mitochondrion) [Panax ginseng] gb|AHJ81036.1| orf105 (mitochondrion) [Panax ginseng]
Niben101Scf04410g09008.1	2.09	16	6820	2	0.02	2	0.02	10	0.12	6	0.06	dentin sialophosphoprotein-related LENGTH=1501
Niben101Scf06662g02024.1	2.70	48	7305	2	0,02	8	0.06	20	0.24	28	0.28	G2484-1 protein, putative isoform 4 [Theobroma cacao] gb|EOY24312.1| G2484-1 protein, putative isoform 4 [Theobroma cacao]
Niben101Scf03225g00012.1	3.18	33	1796	2	0.02	2	0.02	18	0.22	15	0.15	Ubiquitin-associated domain-containing protein 2
Niben101Scf06224g00005.1	2.18	25	957	2	0.02	4	0.03	8	0.10	17	0.17	Unknown protein
Niben101Scf11670g01002.1	2.05	59	1047	2	0.02	30	0.24	25	0.30	34	0.35	Protein CURVATURE THYLAKOID 1B, chloroplastic
Niben101Scf12216g00003.1	2.59	22	4285	2	0.02	2	0.02	10	0.12	12	0.12	ATP binding microtubule motor family protein LENGTH=1051
Niben101Scf04114g00030.1	2.22	45	840	2	0.02	13	0.11	16	0.19	29	0.29	mitovirus RNA-dependent RNA polymerase [Medicago truncatula]
Niben101Scf04114g01080.1	2.16	47	252	2	0.02	12	0.10	11	0.13	36	0.37	Unknown protein
			1510	2	0.02	4	0.03	16	0.19	10	0.10	PH-response transcription factor pacC/RIM101 isoform 1 [Theobroma cacao] gb|EOY06334.1| PH-response

Niben101Scf22171g00007.1	2.30	26										transcription factor pacC/RIM101 isoform 1 [Theobroma cacao]
Niben101Scf03751g01002.1	2.49	21	2093	2	0.02	2	0.02	13	0.16	8	0.08	Polyadenylate-binding protein 1
Niben101Scf02210g09009.1	2.14	12	2583	2	0.02	1	0.01	8	0.10	4	0.04	BnaC09g43760D [Brassica napus]
Niben101Scf05813g00005.1	2.30	37	282	2	0.02	8	0.06	14	0.17	23	0.23	Co-chaperone protein HscB homolog
Niben101Scf02929g03001.1	2.18	25	1077	2	0.02	4	0.03	8	0.10	17	0.17	ORF65d [Pinus koraiensis] gb|ABP35508.1| ORF65d [Pinus koraiensis]
Niben101Scf01521g09001.1	2.68	57	1862	2	0.02	12	0.10	30	0.36	27	0.27	Chain A, N- And C-terminal Helices Of Oat Lov2 (404-546) Are Involved In Light-induced Signal Transduction (cryo Dark Structure Of Lov2 (404-546)) Chain A, N- And C-Terminal Helices Of Oat Lov2 (404-546) Are Involved In Light-Induced Signal Transduction (Cryo- Trapped Light Structure Of Lov2 (404-546))
Niben101Scf00318g10005.1	2.56	40	2904	2	0.02	7	0.06	20	0.24	20	0.20	Omega-3 fatty acid desaturase, chloroplastic
Niben101Scf05929g02002.1	2.16	17	1471	2	0.02	2	0.02	6	0.07	11	0.11	RNA-binding protein 39
Niben101Scf28682g00002.1	2.22	34	501	2	0.02	8	0.06	18	0.22	16	0.16	ATPase subunit 8 (mitochondrion) [Gossypium hirsutum]
Niben101Scf00369g11018.1	2.43	20	983	2	0.02	2	0.02	8	0.10	12	0.12	Translationally-controlled tumor protein homolog
Niben101Scf02569g03009.1	2.30	37	282	2	0.02	8	0.06	14	0.17	23	0.23	Co-chaperone protein HscB homolog
Niben101Scf15187g00005.1	2.043	20	3490	2	0.02	3	0.02	6	0.07	14	0.14	Topoisomerase II-associated protein PAT1 LENGTH=782
Niben101Scf04787g02015.1	2.34	58	1423	2	0.02	18	0.15	38	0.46	20	0.20	Unknown protein
Niben101Scf08804g01015.1	2.43	34	399	2	0.02	6	0.05	18	0.22	16	0.16	Unknown protein
Niben101Scf11408g01031.1	2.09	32	1281	2	0.02	8	0.06	12	0.14	20	0.20	UDP-D-glucuronate 4-epimerase 1 LENGTH=429
			2304	2	0.02	4	0.03	12	0.14	21	0.21	Bromodomain-containing factor 2

Niben101Scf02392g00001.1	2.63	33									
Niben101Scf02576g00011.1	2.19	88	1774	2	0.02	56	0.45	40	0.48	48	0.49	Aquaporin-like superfamily protein LENGTH=285
Niben101Scf01093g04003.1	2.14	12	1882	2	0.02	1	0.01	4	0.05	8	0.08	Protein kinase superfamily protein LENGTH=380
Niben101Scf02164g06013.1	2.16	23	7652	2	0.02	4	0.03	11	0.13	12	0.12	localization and RNA pol II promoter Fmp27 domain protein [Medicago truncatula]
Niben101Scf25246g00003.1	2.30	24	516	2	0.02	2	0.02	20	0.24	4	0.04	orf105a (mitochondrion) [Batis maritima] gb|AIC83344.1| orf105a (mitochondrion) [Batis maritima]
Niben101Scf03510g06011.1	2.46	37	1137	2	0.02	6	0.05	25	0.30	12	0.12	Ethylene-responsive transcription factor 4
Niben101Scf03510g06007.1	2.09	16	789	2	0.02	2	0.02	10	0.12	6	0.06	Unknown protein
Niben101Scf01795g04027.1	2.51	39	390	2	0.02	7	0.06	22	0.26	17	0.17	Rubisco accumulation factor 1, chloroplastic
Niben101Scf03184g06001.1	2.64	16	2645	2	0.02	1	0.01	8	0.10	8	0.08	Zinc finger protein CONSTANS-LIKE 7
Niben101Scf18637g02010.1	2.27	48	4728	2	0.02	15	0.12	22	0.26	26	0.26	global transcription factor group E7 LENGTH=590
Niben101Scf01905g00001.1	2.09	16	1974	2	0.02	2	0.02	6	0.07	10	0.10	Transmembrane 9 superfamily member 11
Niben101Scf00897g03001.1	2.04	24	1614	2	0.02	5	0.04	10	0.12	14	0.14	Glutathione S-transferase 3
Niben101Scf08137g02022.1	2.59	34	4482	2	0.02	4	0.03	24	0.29	10	0.10	Protein argonaute 1B
Niben101Scf13164g00010.1	2.43	20	2367	2	0.02	2	0.02	12	0.14	8	0.08	Importin subunit alpha-1a
Niben101Scf03839g12008.1	2.66	74	716	2	0.02	20	0.16	44	0.53	30	0.30	Organ-specific protein P4
Niben101Scf00150g01014.1	2.17	42	3349	2	0.02	13	0.11	24	0.29	18	0.18	Importin subunit beta-3
Niben101Scf01049g05011.1	3.04	30	4807	2	0.02	2	0.02	16	0.19	14	0.14	Phosphatidylinositol 4-kinase alpha 1
Niben101Scf06277g03006.1	2.04	15	3066	2	0.02	2	0.02	8	0.10	7	0.07	Serine/threonine-protein kinase 4
Niben101Scf00047g02014.1	2.84	40	3948	2	0.02	4	0.03	28	0.34	12	0.12	Phosphoglucan, water dikinase, chloroplastic
Niben101Scf09954g00010.1	2.14	24	2239	2	0.02	4	0.03	16	0.19	8	0.08	Eukaryotic translation initiation factor 3 subunit B
Niben101Scf14098g00005.1	2.33	76	706	2	0.02	34	0.28	44	0.53	32	0.33	Unknown protein

Niben101Scf05419g00006.1	2.04	11	2239	2	0.02	1	0.01	7	0.08	4	0.04	Protein SUPPRESSOR OF GENE SILENCING 3 homolog
Niben101Scf01435g01001.1	2.14	24	2522	2	0.02	4	0.03	16	0.19	8	0.08	purple acid phosphatase 27 LENGTH=611
Niben101Scf04764g10019.1	2.16	23	5562	2	0.02	4	0.03	11	0.13	12	0.12	Zinc finger FYVE domain-containing protein 26
Niben101Scf10579g02007.1	2.09	22	6376	2	0.02	4	0.03	10	0.12	12	0.12	DNA binding protein, putative [Ricinus communis] gb|EEF52043.1| DNA binding protein, putative [Ricinus communis]
Niben101Scf09604g00009.1	2.93	28	3486	2	0.02	2	0.02	12	0.14	16	0.16	Coatomer subunit beta-1
Niben101Scf04767g00007.1	2.09	16	4084	2	0.02	2	0.02	6	0.07	10	0.10	Kinesin-related protein 3
Niben101Scf05012g01001.1	2.09	22	1921	2	0.02	4	0.03	12	0.14	10	0.10	NAD(P)-binding R isoform 4 [Theobroma cacao]
Niben101Scf04738g02001.1	2.80	26	1338	2	0.02	2	0.02	10	0.12	16	0.16	GDSL esterase/lipase
Niben101Scf04738g05019.1	2.09	16	1557	2	0.02	2	0.02	6	0.07	10	0.10	Chaperone protein DnaJ
Niben101Scf01639g03001.1	2.20	24	3688	2	0.02	4	0.03	10	0.12	14	0.14	ETO1-like protein 1
Niben101Scf07253g02020.1	2.02	15	2909	2	0.02	2	0.02	9	0.11	6	0.06	Amine oxidase family member 1
Niben101Scf15244g00004.1	2.14	16	5728	2	0.02	2	0.02	8	0.10	8	0.08	Nuclear receptor corepressor 1
Niben101Scf03049g07019.1	2.14	28	4327	2	0.02	6	0.05	12	0.14	16	0.16	ATP-dependent RNA helicase DED1
Niben101Scf04068g00003.1	2.14	16	1620	1	0.01	4	0.03	8	0.10	8	0.08	TCP family transcription factor LENGTH=401
Niben101Scf02831g05015.1	2.04	11	1692	1	0.01	2	0.02	7	0.08	4	0.04	3-hydroxyisobutyrate dehydrogenase
Niben101Scf07510g00013.1	2.16	28	4577	1	0.01	8	0.06	6	0.07	22	0.22	myosin 2 LENGTH=1220
Niben101Scf02104g00002.1	2.54	18	561	1	0.01	2	0.02	4	0.05	14	0.14	Protein Ycf2
Niben101Scf06291g03020.1	2.59	16	4226	1	0.01	2	0.02	6	0.07	10	0.10	transcription cofactor, putative [Ricinus communis] gb|EEF31930.1| transcription cofactor, putative [Ricinus communis]
Niben101Scf01510g00010.1	2.54	19	390	1	0.01	3	0.02	7	0.08	12	0.12	ATP synthase subunit alpha
Niben101Scf00119g05010.1	2.80	18	1776	1	0.01	2	0.02	8	0.10	10	0.10	Heat shock 70 kDa protein 1A
Niben101Scf01395g05008.1	3.34	26	2942	1	0.01	2	0.02	14	0.17	12	0.12	Outer arm dynein light chain 1 protein LENGTH=1708

Niben101Scf08567g00005.1	2.79	73	762	1	0.01	34	0.28	36	0.43	37	0.38	NADH-ubiquinone oxidoreductase chain 4
Niben101Scf05528g01010.1	2.09	16	1942	1	0.01	4	0.03	6	0.07	10	0.10	RNA-binding KH domain-containing protein LENGTH=315
Niben101Scf08128g00006.1	2.09	22	2705	1	0.01	8	0.06	10	0.12	12	0.12	Eukaryotic translation initiation factor 3 subunit B
Niben101Scf05439g02015.1	4.13	65	438	1	0.01	4	0.03	26	0.31	39	0.40	BnaCnng48510D [Brassica napus]
Niben101Scf05387g09003.1	2.13	16	4283	1	0.01	4	0.03	9	0.11	7	0.07	ATP-dependent RNA helicase DHH1
Niben101Scf00254g00006.1		19	362					15	0.18	4	0.04	heavy metal-associated domain protein [Medicago truncatula]
Niben101Scf01374g13005.1		18	2687					14	0.17	4	0.04	Receptor-like protein kinase
Niben101Scf03738g00006.1		16	2646					10	0.12	6	0.06	plastid movement impaired1 LENGTH=843
Niben101Scf09116g02017.1		12	777					10	0.12	2	0.02	plastid developmental protein DAG, putative LENGTH=229

Table S3. Positions of P-motifs in the NbPPR protein sequence

P-motif number	amino acid position start	amino acid position end
1	133	167
2	168	202
3	203	237
4	238	272
5	273	307
6	308	342
7	383	417
8	418	452

Table S4. Numbers of infection sites in NbPPR-silenced and control leaves. The table summarizes the number of infection sites for each leaf analyzed, with dpi: day post infiltration, rep: replicate, N: number of leaves per replicate, n: number of infection sites .

dpi of tagged virus		VIGS-virus	Rep.	N	n
6dpi	JSBWMV-CPRT:RFP	TRV:00	1	5	21
			2	5	16
			3	6	14
			**total**	**16**	**51**
		TRV:NbPPR	1	5	64
			2	7	0
			3	7	27
			**total**	**19**	**91**
3 dpi	TuMV-GFP	TRV:00	1	2	9
			2	2	2
			3	2	1
			4	5	14
			**total**	**11**	**26**
		TRV:NbPPR	1	2	46
			2	2	5
			3	2	5
			4	4	29
			**total**	**10**	**85**
4 dpi		TRV:00	1	2	6
			2	2	0
			3	2	8
			4	5	17
			**total**	**11**	**31**
		TRV:NbPPR	1	2	31
			2	2	1
			3	2	8
			4	5	17
			**total**	**11**	**57**

Table S5. Positions of RNA binding motif of GFP:NbPPR in plant viral genomes

Family	Genus	Virus	Accession number	Start	End	Match	Length of match	Accuracy (%)	Strand	Target ORF	Function (not exclusive) in
Virgaviridae	Furovirus	JSBWMV RNA1	NC_038850	4367	4373	TATGCGG	7	85.71429	Plus	rep-readthrough	replication
			NC_038850	4571	4577	TATGCGA	7	85.71429	Plus	rep-readthrough	replication
			NC_038850	4651	4657	TATGCAC	7	85.71429	Plus	rep-readthrough	replication
			NC_038850	4809	4815	TATGCTC	7	85.71429	Plus	rep-readthrough	replication
			NC_038850	5749	5755	GATGCGC	7	85.71429	Plus	MP	movement
		JSBWMV RNA2	NC_038851	744	750	AATGCGC	7	85.71429	Plus	CP	encapsidation
			NC_038851	804	810	TATACGC	7	85.71429	Plus	CP	encapsidation
			NC_038851	999	1005	TATGCGG	7	85.71429	Plus	RT	transmission
			NC_038851	1395	1401	TATACGC	7	85.71429	Plus	RT	transmission
			NC_038851	1397	1403	TACGCGC	7	85.71429	Plus	RT	transmission
			NC_038851	1409	1415	TATGCGC	7	100	Plus	RT	transmission
			NC_038851	2913	2919	CATGCGC	7	85.71429	Plus	CRP	silencing suppression
		SBWMV RNA1	NC_002041	479	485	TAAGCGC	7	85.71429	Plus	rep	replication
			NC_002041	652	658	TATACGC	7	85.71429	Plus	rep	replication
			NC_002041	756	762	TATGCGA	7	85.71429	Plus	rep	replication
			NC_002041	1599	1605	TATGTGC	7	85.71429	Plus	rep	replication
			NC_002041	1644	1650	TATGCGG	7	85.71429	Plus	rep	replication
			NC_002041	1683	1689	TATGCAC	7	85.71429	Plus	rep	replication
			NC_002041	2816	2822	TTTGCGC	7	85.71429	Plus	rep	replication
			NC_002041	3247	3253	TATGCGT	7	85.71429	Plus	rep	replication
			NC_002041	3528	3534	TATGCGA	7	85.71429	Plus	rep	replication
			NC_002041	4704	4710	AATGCGC	7	85.71429	Plus	rep-readthrough	replication

	NC_002041	5433	5439	TATGCTC	7	85.71429	Plus	rep-readthrough	replication
	NC_002041	5518	5524	TATGGGC	7	85.71429	Plus	rep-readthrough	replication
SBCMV RNA1	NC_002351	762	768	TATGCGA	7	85.71429	Plus	rep	replication
	NC_002351	1689	1695	TATGCAC	7	85.71429	Plus	rep	replication
	NC_002351	2052	2058	TTTGCGC	7	85.71429	Plus	rep	replication
	NC_002351	2685	2691	TATGCGA	7	85.71429	Plus	rep	replication
	NC_002351	4280	4286	TTTGCGC	7	85.71429	Plus	rep-readthrough	replication
	NC_002351	4484	4490	TATGCGT	7	85.71429	Plus	rep-readthrough	replication
	NC_002351	4674	4680	AATGCGC	7	85.71429	Plus	rep-readthrough	replication
SBCMV RNA2	NC_002330	742	748	AATGCGC	7	85.71429	Plus	CP	encapsidation
	NC_002330	2064	2070	TATGCTC	7	85.71429	Plus	RT	transmission
	NC_002330	2757	2763	TATGGGC	7	85.71429	Plus	CRP	silencing suppression
	NC_002330	2917	2923	CATGCGC	7	85.71429	Plus	CRP	silencing suppression
	NC_002330	3034	3040	TATGCTC	7	85.71429	Plus	CRP	silencing suppression
CWMV RNA1	NC_002359	756	762	TATGCGA	7	85.71429	Plus	rep	replication
	NC_002359	1448	1454	TTTGCGC	7	85.71429	Plus	rep	replication
	NC_002359	1644	1650	TATGCGG	7	85.71429	Plus	rep	replication
	NC_002359	3443	3449	TATGCAC	7	85.71429	Plus	rep	replication
CWMV RNA2	NC_002356	140	146	TATGCGA	7	85.71429	Plus	5'UTR	
	NC_002356	1191	1197	TATGCGG	7	85.71429	Plus	RT	transmission
	NC_002356	2439	2445	AATGCGC	7	85.71429	Plus	RT	transmission
OGSV RNA1	NC_002358	1404	1410	GATGCGC	7	85.71429	Plus	rep	replication
	NC_002358	2546	2552	TATGCGG	7	85.71429	Plus	rep	replication
	NC_002358	3523	3529	TCTGCGC	7	85.71429	Plus	rep	replication
	NC_002358	4604	4610	TATGCGT	7	85.71429	Plus	rep-readthrough	replication
	NC_002358	4842	4848	TACGCGC	7	85.71429	Plus	rep-readthrough	replication
	NC_002358	6122	6128	TATGGGC	7	85.71429	Plus	MP	movement
SCSV RNA1	NC_004014	279	285	TGTGCGC	7	85.71429	Plus	rep	replication
	NC_004014	1734	1740	AATGCGC	7	85.71429	Plus	rep	replication
	NC_004014	2074	2080	TATGCGG	7	85.71429	Plus	rep	replication

		NC_004014	2404	2410	TATCCGC	7	85.71429	Plus	rep	replication
		NC_004014	3412	3418	TATGCGA	7	85.71429	Plus	rep	replication
		NC_004014	4398	4404	CATGCGC	7	85.71429	Plus	rep-readthrough	replication
		NC_004014	5691	5697	TATGCGG	7	85.71429	Plus	MP	movement
		NC_004014	5961	5967	TATGCAC	7	85.71429	Plus	MP	movement
		NC_004014	6745	6751	TAGGCGC	7	85.71429	Plus	3'UTR	
	SCSV RNA2	NC_004015	1228	1234	TATGCGA	7	85.71429	Plus	RT	transmission
		NC_004015	1596	1602	TTTGCGC	7	85.71429	Plus	RT	transmission
		NC_004015	3285	3291	TAGGCGC	7	85.71429	Plus	3'UTR	
Tobamovirus	TMV	NC_001367	2501	2507	TCTGCGC	7	85.71429	Plus	rep	replication
Pecluvirus	PCV RNA1	NC_003672	581	587	AATGCGC	7	85.71429	Plus	rep	replication
		NC_003672	771	777	TATGAGC	7	85.71429	Plus	rep	replication
		NC_003672	1014	1020	TATGAGC	7	85.71429	Plus	rep	replication
		NC_003672	3504	3510	TCTGCGC	7	85.71429	Plus	rep	replication
	PVC RNA2	NC_003668	3437	3443	TTTGCGC	7	85.71429	Plus	TGBp1	movement
Pomovirus	PMTV RNA1	NC_003723	538	544	GAUGCGC	7	85.71429	Plus	rep	replication
		NC_003723	997	1003	UAUGAGC	7	85.71429	Plus	rep	replication
		NC_003723	1777	1783	UAUGAGC	7	85.71429	Plus	rep	replication
		NC_003723	2751	2757	UAUGCGC	7	100	Plus	rep	replication
		NC_003723	4287	4293	UAUGCCC	7	85.71429	Plus	rep-readthrough	replication
		NC_003723	4630	4636	UCUGCGC	7	85.71429	Plus	rep-readthrough	replication
	PMTV RNA2	NC_003725	1657	1663	UUUGCGC	7	85.71429	Plus	TGBp1	movement
	PMTV RNA3	NC_003724	806	812	UAUGAGC	7	85.71429	Plus	endCP	encapsidation
		NC_003724	1463	1469	GAUGCGC	7	85.71429	Plus	RT	transmission
		NC_003724	2114	2120	UAUGCGG	7	85.71429	Plus	RT	transmission
Tobravirus	TRV RNA1	NC_003805	854	860	TATGCGA	7	85.71429	Plus	rep	replication
		NC_003805	1834	1840	TATGCGT	7	85.71429	Plus	rep	replication
		NC_003805	3165	3171	TTTGCGC	7	85.71429	Plus	rep	replication
		NC_003805	5180	5186	CATGCGC	7	85.71429	Plus	rep-readthrough	replication
		NC_003805	5189	5195	TGTGCGC	7	85.71429	Plus	rep-readthrough	replication

			NC_003805	5867	5873	TAGGCGC	7	85.71429	Plus	MP	movement
		TRV RNA2	NC_003811	752	758	TATGAGC	7	85.71429	Plus	CP	encapsidation
			NC_003811	1432	1438	TATTCGC	7	85.71429	Plus	s2GP2	
Potyviridae	Potyvirus	TuMV	NC_002509	1053	1059	TTTGCGC	7	85.71429	Plus	P1-pro	polyprotein processing
			NC_002509	1520	1526	TATGAGC	7	85.71429	Plus	HC-pro	polyprotein processing / silencing suppresionsilencing suppression/ transmission
			NC_002509	2726	2732	TATGCTC	7	85.71429	Plus	P3	virulence factor
			NC_002509	3886	3892	TATGCTC	7	85.71429	Plus	CI	replication / movement
			NC_002509	4216	4222	GATGCGC	7	85.71429	Plus	CI	replication / movement
			NC_002509	4988	4994	TATGGGC	7	85.71429	Plus	CI	replication / movement
			NC_002509	5240	5246	TATGCAC	7	85.71429	Plus	CI	replication / movement
			NC_002509	8508	8514	TCTGCGC	7	85.71429	Plus	Nib	replication
			NC_002509	8980	8986	GATGCGC	7	85.71429	Plus	CP	encapsidation
	Bymovirus	WYMV RNA1	NC_002350	1094	1100	UAUGGGC	7	85.71429	Plus	P3	virulence factor
			NC_002350	2878	2884	CAUGCGC	7	85.71429	Plus	CI	replication / movement
			NC_002350	3594	3600	GAUGCGC	7	85.71429	Plus	6K2	Membrane rearrangement, VRC anchoring
			NC_002350	4450	4456	AAUGCGC	7	85.71429	Plus	Nia-Pro	polyprotein processing
			NC_002350	5408	5414	UUUGCGC	7	85.71429	Plus	NIb	replication
			NC_002350	5585	5591	UAUUCGC	7	85.71429	Plus	Nib	replication
			NC_002350	5629	5635	UAUGCGA	7	85.71429	Plus	Nib	replication
			NC_002350	6006	6012	UAUGCUC	7	85.71429	Plus	Nib	replication
			NC_002350	6355	6361	UAUGCGU	7	85.71429	Plus	Nib	replication
			NC_002350	7497	7503	UAUGCAC	7	85.71429	Plus	3'UTR	
		WYMV RNA2	NC_002349	473	479	UAUGCGU	7	85.71429	Plus	HC-Pro	polyprotein processing / silencing

										suppresionsilencing suppression/
			NC_002349	1227	1233	UUUGCGC	7	85.71429	Plus	CP-RT	transmission?
		BaYMV RNA1	NC_002990	140	146	UUUGCGC	7	85.71429	Plus	5'UTR	
			NC_002990	435	441	UCUGCGC	7	85.71429	Plus	P3	virulence
			NC_002990	579	585	GAUGCGC	7	85.71429	Plus	P3	virulence
			NC_002990	745	751	UAUGGGC	7	85.71429	Plus	P3	virulence
			NC_002990	1552	1558	UAUGCAC	7	85.71429	Plus	CI	replication / movement
			NC_002990	1754	1760	UUUGCGC	7	85.71429	Plus	CI	replication / movement
			NC_002990	1792	1798	UAUGGGC	7	85.71429	Plus	CI	replication / movement
			NC_002990	1855	1861	UCUGCGC	7	85.71429	Plus	CI	replication / movement
			NC_002990	2379	2385	AAUGCGC	7	85.71429	Plus	CI	replication / movement
			NC_002990	2890	2896	UAUGCGC	7	100	Plus	CI	replication / movement
			NC_002990	5188	5194	UAUGCGC	7	100	Plus	Nib	replication
			NC_002990	5287	5293	UAUGCGA	7	85.71429	Plus	Nib	replication
			NC_002990	5814	5820	UAUUCGC	7	85.71429	Plus	Nib	replication
			NC_002990	6529	6535	GAUGCGC	7	85.71429	Plus	CP	encapsidation
			NC_002990	6912	6918	UGUGCGC	7	85.71429	Plus	CP	encapsidation
			NC_002990	7119	7125	CAUGCGC	7	85.71429	Plus	CP	encapsidation
		BaYMV RNA2	NC_002991	462	468	UGUGCGC	7	85,71429	Plus	P1	polyprotein processing
			NC_002991	510	516	UGUGCGC	7	85.71429	Plus	P1	polyprotein processing
Benyviridae	Benyvirus	BNYVV RNA1	NC_003514	277	283	UAUGCUC	7	85.71	Plus	rep	replication
			NC_003514	1159	1165	UAUGCGC	7	100.00	Plus	rep	replication
			NC_003514	4988	4994	AAUGCGC	7	85.71	Plus	rep	replication
		BNYVV RNA2	NC_003515	571	577	UAUACGC	7	85.71	Plus	CP	encapsidation
			NC_003515	1137	1143	UAUGCGU	7	85.71	Plus	RT	transmission
			NC_003515	3741	3747	UAUGCGG	7	85.71	Plus	TGBp3	movement
		BNYVV RNA3	NC_003516	247	253	UAUGCGU	7	85.71	Plus	5'UTR	
			NC_003516	458	464	UAGGCGC	7	85.71	Plus	P25	virulence
			NC_003516	1422	1428	UAUGCGA	7	85.71	Plus	P25	virulence

BNYVV RNA5	NC_003513	177	183	UAUGCCC	7	85.71	Plus	5'UTR
	NC_003513	1145	1151	UAUGCGG	7	85.71	Plus	3'UTR

Table S6. Numbers of reads and normalized numbers of reads per replicate and condition for each scaffold identified in RIP experiments as bound to GFP:NbPPR

		GFP				GFP:NbPPR				
		Rep1		Rep2		Rep1		Rep2		
Gene / Scaffold	Scaffold length	Reads	Counts per million	Reads	Counts per million	Reads	Counts per million	Reads	Counts per million	Annotation
Niben101Scf04451g00027.1	246	155	1.92	434	3.51	1005	12.06	968	9.84	Mitochondrial protein, putative [Medicago truncatula]
Niben101Scf05689g02012.1	429	122	1.51	406	3.29	1260	15.12	1220	12.40	NADH-quinone oxidoreductase subunit H
Niben101Scf15885g00035.1	852	120	1.49	487	3.94	1504	18.04	1163	11.82	conserved hypothetical protein [Ricinus communis] gb|EEF25773.1| conserved hypothetical protein [Ricinus communis]
Niben101Scf02309g03017.1	378	73	0.91	305	2.47	714	8.57	636	6.47	conserved hypothetical protein [Ricinus communis] gb|EEF25773.1| conserved hypothetical protein [Ricinus communis]
Niben101Scf09696g01037.1	420	16	0.2	51	0.41	118	1.42	123	1.25	conserved hypothetical protein [Ricinus communis] gb|EEF25773.1| conserved hypothetical protein [Ricinus communis]
Niben101Scf01681g02009.1	3393	110	1.37	119	0.96	584	7.01	402	4.09	Protein IQ-DOMAIN 14
Niben101Scf08162g00010.1	300	93	1.15	276	2.23	1072	12.86	972	9.88	NADH-quinone oxidoreductase subunit H 1
Niben101Scf04432g06003.1	345	91	1.13	398	3.22	793	9.51	633	6.43	conserved hypothetical protein [Ricinus communis] gb|EEF45986.1| conserved

									hypothetical protein [Ricinus communis]
Niben101Scf05540g00010.1	345	56	0.7	262	2.12	569	6.83	436	4.43	conserved hypothetical protein [Ricinus communis] gb|EEF45986.1| conserved hypothetical protein [Ricinus communis]
Niben101Scf11347g00005.1	879	82	1.02	259	2.10	675	8.10	636	6.47	NADH-quinone oxidoreductase subunit H
Niben101Scf04048g00015.1	687	81	1.01	263	2.13	908	10.89	690	7.01	orf109 (mitochondrion) [Panax ginseng] gb|AHJ81034.1| orf109 (mitochondrion) [Panax ginseng]
Niben101Scf00367g07004.1	648	33	0.41	118	0.96	519	6.23	358	3.64	orf109 (mitochondrion) [Panax ginseng] gb|AHJ81034.1| orf109 (mitochondrion) [Panax ginseng]
Niben101Scf06928g00013.1	279	24	0.3	75	0.61	410	4.92	277	2.82	orf109 (mitochondrion) [Panax ginseng] gb|AHJ81034.1| orf109 (mitochondrion) [Panax ginseng]
Niben101Scf14482g00003.1	459	4	0.05	20	0.16	80	0.96	57	0.58	orf109 (mitochondrion) [Panax ginseng] gb|AHJ81034.1| orf109 (mitochondrion) [Panax ginseng]
Niben101Scf00854g06020.1	887	52	0.65	152	1.23	330	3.96	400	4.07	Unknown protein
Niben101Scf01184g09019.1	3270	45	0.56	93	0.75	259	3.11	217	2.21	hero resistance protein 3 homologue [Solanum lycopersicum]
Niben101Scf02074g06077.1	570	41	0.51	34	0.28	158	1.90	138	1.40	NADH dehydrogenase subunit 2 (mitochondrion) [Isoetes engelmannii]
Niben101Scf02074g01039.1	303	33	0.41	38	0.31	204	2.45	122	1.24	Ribosomal protein S10 [Medicago truncatula]
Niben101Scf03515g00078.1	1333	27	0.34	47	0.38	143	1.72	166	1.69	NADH-quinone oxidoreductase subunit D
Niben101Scf00916g01010.1	6666	26	0.32	22	0.18	124	1.49	64	0.65	Unknown protein
Niben101Scf00482g08002.1	204	25	0.31	111	0.90	277	3.32	219	2.23	Unknown protein

Niben101Scf14427g00008.1	1044	24	0.3	52	0.42	143	1.72	129	1.31	DNA polymerase (mitochondrion) [Silene vulgaris]
Niben101Scf05250g00010.1	7708	20	0.25	27	0.22	143	1.72	64	0.65	Unknown protein
Niben101Scf03515g00058.1	339	19	0.24	58	0.47	140	1.68	168	1.71	BnaUnng03880D, partial [Brassica napus]
Niben101Scf02041g00002.1	1315	18	0.22	32	0.26	101	1.21	103	1.05	Chitinase 8
Niben101Scf00849g01019.1	414	18	0.22	49	0.40	108	1.30	120	1.22	NADH-ubiquinone oxidoreductase chain 4
Niben101Scf03431g00004.1	453	18	0.22	47	0.38	97	1.16	118	1.20	NADH-quinone oxidoreductase subunit H
Niben101Scf18129g00003.1	1125	17	0.21	22	0.18	88	1.06	95	0.97	NADH-quinone oxidoreductase subunit D
Niben101Scf10881g00025.1	660	16	0.2	54	0.44	145	1.74	103	1.05	NADH-ubiquinone oxidoreductase chain, putative [Ricinus communis] gb|EEF27701.1| NADH-ubiquinone oxidoreductase chain, putative [Ricinus communis]
Niben101Scf14482g00006.1	285	14	0.17	16	0.13	67	0.80	52	0.5	BnaCnng48510D [Brassica napus]
Niben101Scf18129g00016.1	381	11	0.14	24	0.19	136	1.63	119	1.2	BnaCnng48510D [Brassica napus]
Niben101Scf08015g02002.1	249	12	0.15	34	0.28	78	0.94	79	0.8	cytochrome c biogenesis FN (mitochondrion) [Hevea brasiliensis]
Niben101Scf05439g02038.1	378	12	0.15	12	0.10	86	1.03	39	0.4	orf116d (mitochondrion) [Batis maritima] gb|AIC83424.1| orf116d (mitochondrion) [Batis maritima]
Niben101Scf04114g01046.1	885	11	0.14	31	0.25	72	0.86	69	0.7	NADH dehydrogenase subunit 5 (mitochondrion) [Silene vulgaris]
Niben101Scf03431g00012.1	306	11	0.14	54	0.44	107	1.28	87	0.9	4-coumarate--CoA ligase-like 7 [Morus notabilis] gb|EXC31783.1| 4-coumarate--CoA ligase-like 7 [Morus notabilis]

Niben101Scf00853g03001.1	213	11	0.14	33	0.27	93	1.12	59	0.6	orf34 gene product (mitochondrion) [Daucus carota subsp. sativus] gb|AEY81172.1| orf34 (mitochondrion) [Daucus carota subsp. sativus]
Niben101Scf02749g04008.1	627	10	0.12	38	0.31	97	1.16	81	0.8	Germin-like protein subfamily 3 member 3
Niben101Scf01374g02004.1	693	8	0.1	37	0.30	124	1.49	90	0.9	Cytochrome C assembly protein LENGTH=256
Niben101Scf01745g07016.1	693	8	0.1	37	0.30	134	1.61	96	1.0	Cytochrome C assembly protein LENGTH=256
Niben101Scf02497g07001.1	693	8	0.1	27	0.22	93	1.12	64	0.7	Cytochrome C assembly protein LENGTH=256
Niben101Scf00485g00013.1	693	8	0.1	33	0.27	108	1.30	82	0.8	Cytochrome C assembly protein LENGTH=256
Niben101Scf02002g01024.1	693	6	0.07	37	0.30	130	1.56	94	1.0	Cytochrome C assembly protein LENGTH=256
Niben101Scf00107g03008.1	771	6	0.07	19	0.15	102	1.22	51	0.5	Cysteine-rich venom protein
Niben101Scf15885g00031.1	516	6	0.07	21	0.17	59	0.71	50	0.5	NADH-quinone oxidoreductase subunit D
Niben101Scf00914g03002.1	195	6	0.07	39	0.32	86	1.03	66	0.7	30S ribosomal protein S3

Supplementary methods

Prediction of target signal

The "TargetP2.0"-tool employs protein-sequences and the prediction was made for the organism group plant with default parameters. The website is available under https://services.healthtech.dtu.dk/service.php?TargetP-2.0 (retrieval date 08.03.2022 (Almagro Armenteros *et al.* 2019)).

Identification of PPR-motif

PPR.plantenergy (https://ppr.plantenergy.uwa.edu.au/ version: 1f9e982, retrieval date: 07.03.2022 (Cheng *et al.* 2016; Gutmann *et al.* 2020)) was used to identify possible PPR motifs present in the identified PPR sequence. The tool "search for PPRs" without changes in the standard search-parameters was applied. For an overview of the P-motifs of the PPR-sequence, the drawing tool "mydomains" from the prosite.expasy website (https://prosite.expasy.org/cgibin/prosite/mydomains/; retrieval date 07.03.2022) was used (Hulo *et al.* 2008).

Infection with JSBWMV-CP-RT:RFP

Infection with JSBWMV-CPRT:RFP was performed with infectious RNA. Infectious RNA was synthesized from plasmids. pJS1 carrying RNA1 and pJS2 carrying RNA2 of JSBWMV was published in (Yamamiya and Shirako, 2000), pJS2 was modified to express a fluorescent protein-tagged CP-RT (Sprotte et al., Protoplasma, accepted). The plasmids pJS1 and pJS2-CPRT:RFP were linearized with SPE1-HF® (NEB) and then purified with the NucleoSpin Gel and PCR Clean-up kit (Macherey-Nagel) according to the manufacturer's protocol before use as RNA-synthesis template. pJS1 and pJS2-CPRT:RFP harbor an SP6-promotor for RNA synthesis, which was performed with the SP6 RiboMAX™ Large Scale RNA Production System (Promega) according to the manufacturer's instructions with minor changes. The rNTP mix was changed to a concentration of 5 mM ATP, CTP, UTP, and 0.6 mM GTP and 2.67 mM m7G-cap analog (Promega) in the reaction mix. For inoculation of infectious viral RNA, transcribed RNA1^{JSBWMV} and RNA2$^{JSBWMV\text{-}CPRT:RFP}$ were mixed equally and diluted 1:4 with inoculation buffer (50 mM glycine; 50 mM K2HPO4 (pH 9.2)). Celite was used to powder the leaves of *N. benthamiana*. The RNA mixture was distributed in little drops on the upper side of the leaves and then carefully rub inoculated onto the leaf surface. The leaves were rinsed with water after 20 min.
